# Construction of an octosyl acid backbone catalyzed by a radical *S*-adenosylmethionine enzyme and a phosphatase in the biosynthesis of high-carbon sugar nucleoside antibiotics†
†Electronic supplementary information (ESI) available: Fig. S1–S26, Scheme S1, Tables S1–S3, Methods S1–S9, full experimental details, procedures and supplementary references. See DOI: 10.1039/c6sc01826b



**DOI:** 10.1039/c6sc01826b

**Published:** 2016-08-19

**Authors:** Nisha He, Pan Wu, Yongxing Lei, Baofu Xu, Xiaochen Zhu, Gudan Xu, Yaojie Gao, Jianzhao Qi, Zixin Deng, Gongli Tang, Wenqing Chen, Youli Xiao

**Affiliations:** a Key Laboratory of Combinatorial Biosynthesis and Drug Discovery , Ministry of Education , School of Pharmaceutical Sciences , Wuhan University , Wuhan 430071 , China . Email: wqchen@whu.edu.cn; b CAS Key Laboratory of Synthetic Biology , CAS Center for Excellence in Molecular Plant Sciences , Institute of Plant Physiology and Ecology , Shanghai Institutes for Biological Sciences , Chinese Academy of Sciences , 300 FengLin Road , Shanghai 200032 , China . Email: ylxiao@sibs.ac.cn; c University of Chinese Academy of Sciences , Beijing 100039 , China; d State Key Laboratory of Bio-organic and Natural Products Chemistry , Shanghai Institute of Organic Chemistry , Chinese Academy of Sciences , 345 Lingling Road , Shanghai 200032 , China

## Abstract

Unique bicyclic octosyl uronic acid nucleosides include ezomycin, malayamycin, and octosyl acid (OA). They are structurally characterized by OA, an unusual 8-carbon furanosyl nucleoside core proposed to be the precursor to polyoxin and nikkomycin. Despite the well-known bioactivity of these nucleoside antibiotics, the biosynthesis of OA has not been elucidated yet. Here we report the two pivotal enzymatic steps in the polyoxin biosynthetic pathway leading to the identification of OA as a key intermediate. Our data suggest that this intermediate is formed *via* a free radical reaction catalyzed by the radical *S*-adenosylmethionine (SAM) enzyme, PolH, and using 3′-enolpyruvyl uridine 5′-monophosphate (3′-EUMP) as a substrate. Subsequent dephosphorylation catalyzed by phosphatase PolJ converts the resulting octosyl acid 5′-phosphate (OAP) to OA. These results provide, for the first time, significant *in vitro* evidence for the biosynthetic origins of the C8 backbone of OA.

## Abstract 2

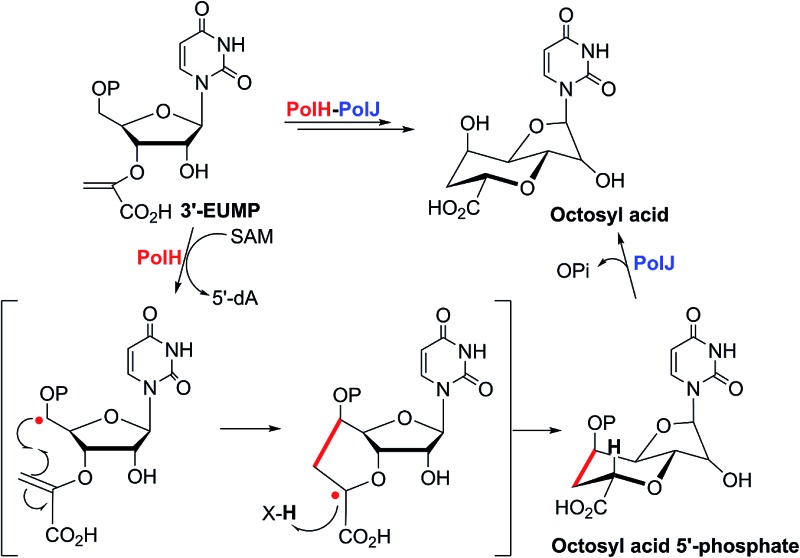
This work provides, for the first time, significant *in vitro* evidence for the biosynthetic origins of octosyl acid through free radical and dephosphorylation enzymatic reactions.

## Introduction

**Fig. 1:**
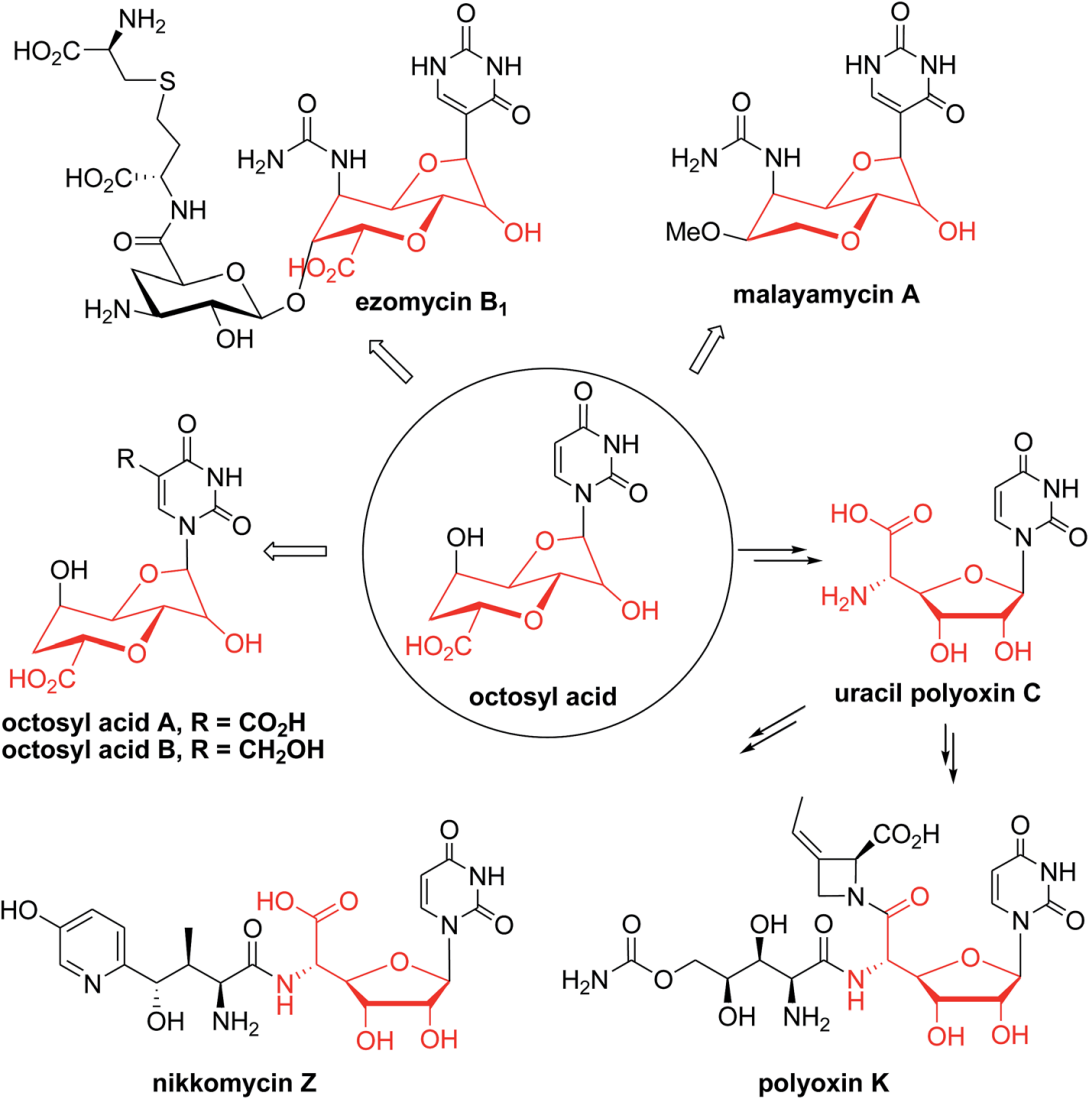
Selected examples of nucleoside antibiotics containing or originating from the octosyl acid (OA) scaffold.

**Scheme 1:**
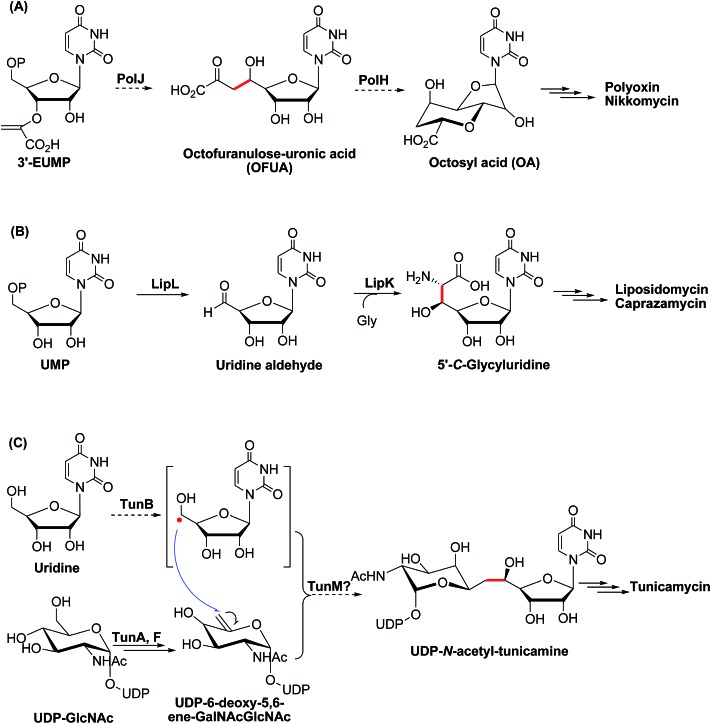
Proposed or characterized biosynthetic pathway for building the high-carbon sugar nucleoside skeleton.

Nucleoside antibiotics constitute more than 200 structurally unique natural products with a wide range of bioactivities, such as antibacterial, antifungal, antitumor, and antiviral activities.^1^ This family of antibiotics is usually biosynthesized by sequential modifications of the nucleoside core scaffold.^1^ The unique bicyclic uronic acid nucleosides, which include ezomycin,^2*a*^ malayamycin,^2*b*^ and octosyl acid (OA),^2*c*^ are structurally characterized by OA, an unusual 8-carbon furanosyl nucleoside skeleton (Fig. 1). The formation of the high carbon sugar (8C's) nucleoside scaffold, OA, has been proposed as a key step in the biosynthesis of polyoxin and nikkomycin and as the precursor to the second key intermediate, aminohexuronic acid (AHA, uracil polyoxin C) (Fig. 1 and Scheme 1A).^1e,3,4^ Polyoxin, a group of structurally-related peptidyl nucleoside antibiotics, is produced by *Streptomyces cacaoi* var. *asoensis* and *Streptomyces aureochromogenes*.^4,5^ The structure of polyoxin resembles UDP-*N*-acetylglucosamine, a building block for fungal chitin biosynthesis, and it thus acts as a potent chitin synthetase inhibitor by targeting fungal cell wall biosynthesis.^6*a*^ Polyoxin has been extensively used as an important fungicide in agriculture and as a potential lead for mammalian antifungal drug development.^6*b*^ Structurally, polyoxin consists of three building blocks including a nucleoside skeleton and two nonproteinogenic amino acids involving polyoximic acid and carbamoylpolyoxamic acid.^1*a*^

Besides the OA skeleton derived natural products including polyoxin, nikkomycin,^7*a*^ malayamycin,^2*b*^ and ezomycin,^2*a*^ only a few high-carbon (>6C's) sugar nucleoside antibiotics have been identified, such as liposidomycin,^7*b*^ caprazamycin,^7*c*^ and tunicamycin^7*d*^ (ESI, Fig. S1†). However, little is known about how the high-carbon sugar scaffolds are biosynthesized, except for 5′-*C*-glycyluridine, which is a key intermediate in the biosynthesis of liposidomycin and has been characterized and shown to be generated from uridine-5′-monophosphate (UMP), consecutively catalyzed by LipL and LipK, a non-heme iron(ii)-dependent dioxygenase and a PLP-dependent transaldolase, respectively (Scheme 1B).^8^ In the biosynthesis of tunicamycin, TunB, a putative radical SAM-dependent protein with its partner TunM, is predicted to catalyze the formation of the C–C bond between the C-5 of the uridyl moiety and the C-6 of the central sugar, *exo*-glycal, which is enzymatically produced by TunA and TunF in a recent revised biosynthesis pathway (Scheme 1C).^9^

Since the identification of OA as the metabolite from the polyoxin producer, *Streptomyces cacaoi* var. *asoensis*, in the 1970s,^2*c*^ discoveries of this unique family of natural products or derivatives have been continuously reported.^1^ To our knowledge, how OA is constructed by nature has remained elusive for almost half a century. Herein, we report the genetic evidence and *in vitro* functional characterization of two enzymes, PolH and PolJ, involved in the early stage of polyoxin biosynthesis. On the basis of the detailed investigation and biochemical analysis of these two consecutive enzymatic reactions, we revised the pathway by identifying OA as a key intermediate.

## Results and discussion

### The genetic role of *polJ* and *polH* in the biosynthesis of polyoxin

Early feeding experiments using [U-^14^C]-uridine, [1-^13^C]-glucose, [3-^14^C]-pyruvate, [3-^14^C]-glycerate,^3^ and [6-^13^C]-glucose^10^ showed that uridine is incorporated intact and the C-3 of pyruvate or glycerate is the source of the C-6′ of OA during the biosynthesis of polyoxin. In addition, the orthologous enzymes encoded by *nikO* ^11^ and *polA* ^4^ have been shown to catalyze the formation of 3′-enoylpyruvaoyl-UMP (3′-EUMP) from PEP and UMP. Taken together, these observations allowed us to propose a preliminary biosynthetic pathway for OA, which consisted of two key steps: (i) the oxidative dephosphorylation of 3′-EUMP catalyzed by PolJ, and (ii) the cyclization of octofuranulose-uronic acid (OFUA) to OA through a radical reaction catalyzed by PolH (Scheme 1A).^4^

To evaluate the roles of *polH* and *polJ* in the proposed biosynthetic pathway, we individually introduced the target mutant cosmids pJTU4774/Δ*polH* and pJTU4774/Δ*polJ* into the *S. aureochromogenes* CXR14 strain (ESI, Fig. S2, Tables S1 and S2, and Methods S2†). A bioassay showed that the metabolites of the target *polH* and *polJ* mutants lacked bioactivity against the indicator fungi, indicating that they lacked the capability to produce polyoxin (ESI, Fig. S3A†). An accumulated metabolite, designated compound X, from the media of the *polH* deletion strain, was furthermore structurally characterized by LC-MS and NMR analysis (ESI, Fig. S3B and C, S4–S9 and Methods S3†). We subsequently analyzed the metabolites of the CXR14::pJTU4774/Δ*polJ* strain, and there were no obvious new metabolite-profiles on the basis of the LC-MS analysis (ESI, Fig. S3B†). These gene knockout experiments demonstrated that *polJ* and *polH* are required for the biosynthesis of polyoxin, but their enzymatic functions remained unexplored.

### *In vitro* characterization of PolJ and PolH

**Fig. 2:**
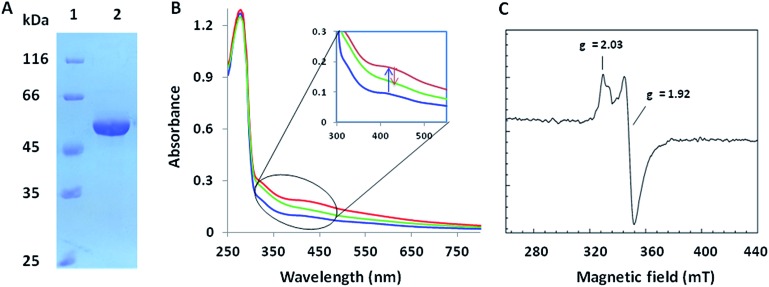
Biochemical characterization of PolH. (A) SDS-PAGE of the anaerobically purified PolH; (B) UV-Vis spectra of the as-isolated (blue), reconstituted (red), and partially reduced (green) PolH; (C) EPR spectrum of reconstituted PolH reduced by sodium dithionite.

We investigated the proposed pathway with *in vitro* characterization of the reactions catalyzed by PolH and PolJ. Based on bioinformatic analysis, PolJ (ESI, Fig. S10†) and PolH (ESI, Fig. S11†) had been assigned as a phosphatase and a radical SAM protein, respectively.^4^ The *polJ* and *polH* genes were individually cloned into pET28a with N-terminal His6 tags followed by protein over-expression in *E. coli*. PolJ was purified to near homogeneity with partial protein contamination due to issues with poor solubility and weak expression (ESI, Fig. S12†). PolH, with the characteristic C_225_XXXC_229_XXC_232_ radical SAM protein motif,^12^ was purified anaerobically and contained a low concentration of iron/sulfur clusters, as evident from the UV-Vis spectrum (Fig. 2A and B). The PolH was reconstituted with enhanced iron/sulfur content and contained one [4Fe–4S] cluster per protein based on the iron^13*a*^ and sulfur^13*b*^ analyses, which indicated that the reconstituted PolH has 3.9 iron ions and 3.2 sulfur atoms per PolH monomer compared to 1.1 iron ion and 1.0 sulfur atom per as-isolated PolH (ESI, Fig. S13†). Electron paramagnetic resonance (EPR) characterization of the as-isolated PolH displayed a typical signal with a *g* value of 2.00 for a [3Fe–4S]^+^ cluster (ESI, Fig. S14A†). After reconstitution and reduction, a typical [4Fe–4S]^+^ cluster feature was observed with an axial EPR signal with *g*_∥_ = 2.03, and *g*_⊥_ = 1.92 (Fig. 2C). Moreover, the same *g*-values were also determined with less intensity from the as-isolated PolH in the presence of a dithionite reductant (ESI, Fig. S14B†). These EPR signals are in agreement with those of other radical-AdoMet [4Fe–4S]^+^ clusters.^14^

### The biochemical activity of PolH and PolJ

Initially, we tried to optimize a variety of reaction conditions for PolH using compound X as a substrate, but unfortunately, our test of the PolH activity yielded negative results, suggesting that it's not a correct prime substrate for PolH. It is probable that compound X is originally synthesized from the very substrate of PolH *via* a pathway independent of polyoxin biosynthesis. We then were motivated to reinvestigate the initial steps leading to the nucleoside skeleton of polyoxin, which are proposed to be governed by PolJ and PolH in our previous report.^4^ As a consequence, the key substrate 3′-EUMP was prepared chemo-enzymatically by PolA's homolog, NikO, and purified by HPLC (ESI, Fig. S15 and Methods S4†). 3′-EUMP was first incubated with PolJ, and the reaction mixtures were analyzed by LC-MS/MS. 3′-Enolpyruvyl-uridine (3′-EU) was determined by tandem MS analysis as the actual dephosphorylation product instead of the proposed oxidative dephosphorylation product, OFUA (ESI, Fig. S16†). Chemically, the C-5′ of 3′-EU(MP) has to be activated as well as dephosphorylation for conversion to OFUA. Enzymatically, the activation of the C-5′ of uridine analogues could be achieved either by an oxidase, like LipL,^8*a*^ with the formation of the aldehyde in the biosynthesis of liposidomycin, or through a radical mechanism, like TunB,^9^ predicted in the biosynthesis of tunicamycin (Scheme 1B and C). Considering the observed net dephosphorylation function and bioinformatic analysis of PolJ as a putative phosphatase,^4^ the proposed transformation of 3′-EUMP to OFUA catalyzed by PolJ would require another enzyme. To test this hypothesis, a PolJ–PolH coupled enzymatic assay was performed either to initiate the reaction with the radical or to push the reaction forward by consumption of the potential intermediates generated by PolJ. The reconstituted PolH plus PolJ were incubated anaerobically with 3′-EUMP with the presence of SAM and dithionite as the general radical SAM enzymatic assay conditions (ESI, Methods S7 and S9†).^14^ The reaction mixture was analyzed by LC-MS/MS, and compared with the purely PolJ catalyzed reaction; the substrate 3′-EUMP with a retention time of 10.2 min was consumed completely and the dephosphorylated 3′-EU was observed at *t*_R_ = 4.05 min. In addition, two new signals at *t*_R_ = 9.89 min and *t*_R_ = 3.67 min with the same molecular mass of 393.03 (designated as an isomer of 3′-EUMP) and 313.06 (designated as an isomer of 3′-EU) as 3′-EUMP and 3′-UM, respectively, were detected (ESI, Fig. S17A and B†). Further comparative tandem MS/MS analysis of these two peaks indicated that the two isomers shared a common uridine motif as 3′-EUMP and 3′-EU with the same ion peaks at *m*/*z* 111.02 (ESI, Fig. S17C and D†). By omitting PolJ in the coupling assay, only the product, the isomer of 3′-EUMP, was detected in the presence of PolH as shown by the LC-MS/MS spectrum. From the above studies, it's most likely that 3′-EUMP was converted by PolH to its isomer.

**Fig. 3:**
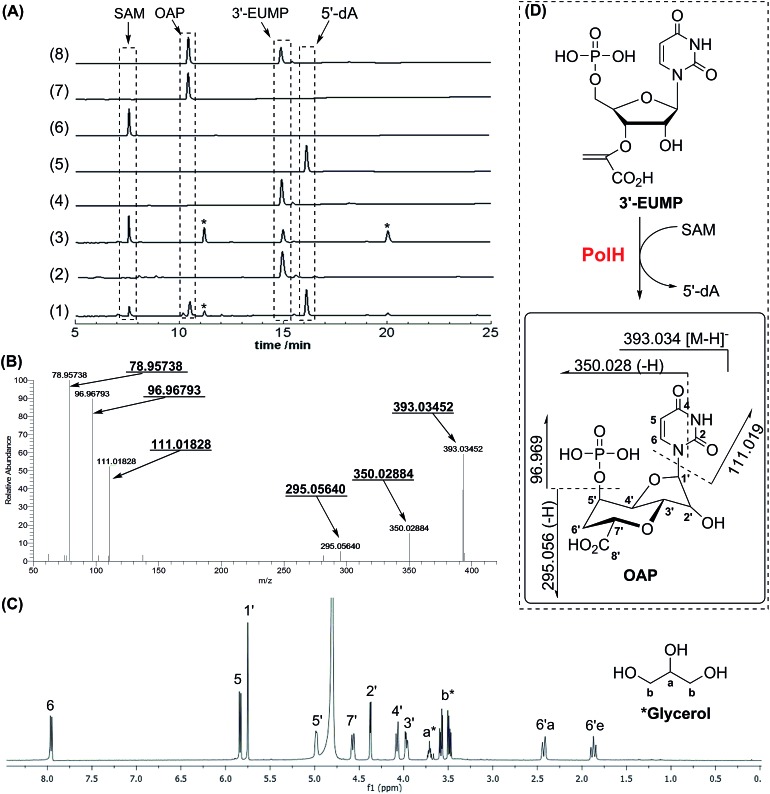
Characterization of the reaction of 3′-EUMP catalyzed by reconstituted PolH. (A) HPLC analysis of the activity assay (monitoring at 260 nm). (1) PolH with 3′-EUMP, SAM and dithionite; (2) without SAM; (3) with denatured PolH; (4) 3′-EUMP standard; (5) 5′-dA standard; (6) SAM standard; (7) purified PolH reaction product (OAP); (8) co-injection of OAP with 3′-EUMP; (B) ESI-MS/MS (78.957, 96.967, 111.018, 295.056, 350.028, and 393.034 (from left to right)); (C) ^1^H-NMR of the purified product (OAP) from the PolH reaction; (D) the reaction scheme of PolH. *The impurities from background reactions in the HPLC assay or contamination from glycerol in the ^1^H-NMR sample.

To elucidate the isomer of 3′-EUMP catalyzed by PolH, a HPLC assay with different control experiments was performed (ESI, Methods S9†). The new product was only generated in the presence of both SAM and sodium dithionite in comparison with the control and standard traces (Fig. 3A1–8, ESI and Fig. S18†). The product was further purified by HPLC and fully characterized by HR-MS and NMR (Fig. 3B and C, ESI and Fig. S18–S23†). The *m*/*z* 393.03 ratio of the purified product determined by ESI-MS/MS analysis was the same as 3′-EUMP with a common ion peak at *m*/*z* 111.02, but different ion peaks at *m*/*z* 295.05 to 305.02 of 3′-EUMP, which may be due to a cyclization reaction of a pyruvate side chain to the nucleoside sugar group (Fig. 3B, ESI and Fig. S18B†). There are 10 and 12 signals in the ^1^H and ^13^C-NMR spectrum, respectively, except for the extra signals from glycerol contamination (ESI, Fig. S19 and S20†). The majority of the peaks in the ^1^H-NMR spectrum were highly consistent with the chemically synthesized standard octosyl acid A (5-carboxylic octosyl acid).^15^ ^1^H–^1^H COSY analysis indicated that C-5′ and C-6′ were continuously connected (ESI, Fig. S21†). HMBC analysis suggested a correlation between the H-1′ with C-4′, H-3′ with C-7′, and H-2′ with C-4′, and with further DEPT and HMQC results, a bicyclic high carbon sugar skeleton OAP structure was unambiguously determined (ESI, Fig. S19 and S21–S23†). Thus, the biosynthetic pathway for OA was consequently revised (ESI, Scheme S1 and Fig. S24†).

### The PolH reaction follows a radical reaction mechanism

**Fig. 4:**
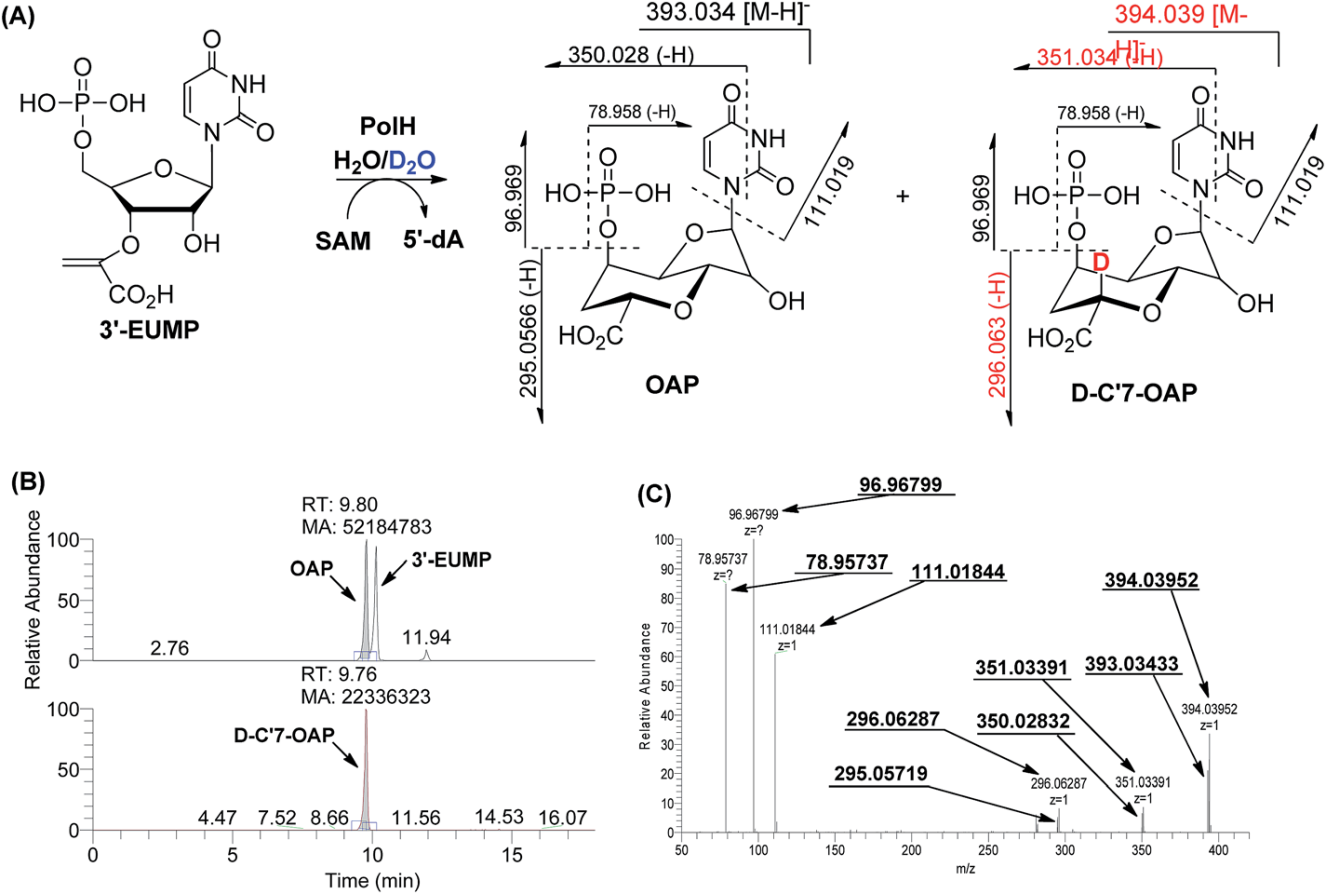
Characterization of the reaction of 3′-EUMP catalyzed by reconstituted PolH in 60% D_2_O containing buffer. (A) Reaction scheme; (B) LC-MS; (C) ESI-MS/MS (78.957, 96.967, 111.018, 295.057, 296.062, 350.028, 351.033, 393.034, and 394.039 (from left to right)).

During the cyclization reaction catalyzed by PolH, the amount of OAP and 5′-dA was increased simultaneously based on the time course monitoring experiments, indicating that SAM was being used as a co-substrate (ESI, Fig. S25†). Therefore, 5′-dA was a co-product instead of a catalytic source for the regeneration of 5′-dA radicals like the well characterized lysine-2,3-aminomutase (LAM).^16^ Presumably, the substrate radical intermediate generated from 3′-EUMP abstracted a hydrogen atom from an amino acid residue with solvent exchangeable ability, as is the case with NeoN as opposed to 5′-dA in the biosynthesis of neomycin.^17^ To verify this hypothesis, the PolH assay was carried out in a 60% deuterium oxide buffer with the clear detection of *m*/*z* 396.05 against *m*/*z* 395.05 by LC-MS/MS for the unlabeled OAP (Fig. 4, ESI and Fig. S26†).

**Scheme 2:**
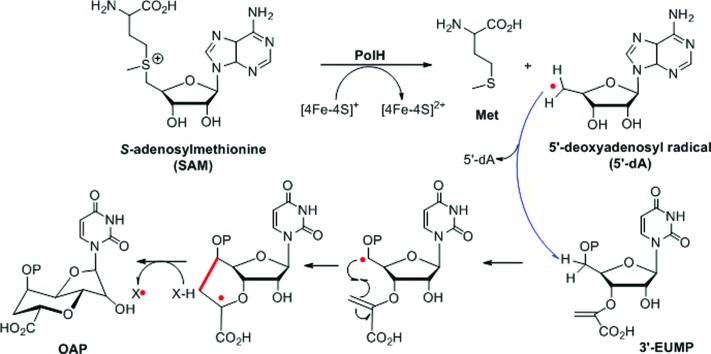
The proposed reaction mechanism catalyzed by PolH.

Given the above results, the PolH reaction mechanism is described in Scheme 2 which aligns well with the previous isotopic feeding experiments about the carbon sources of C-5′ and C-6′.^3,10^ We thus propose that the 5′-dA radical, generated from SAM and catalyzed by the reduced [4Fe–4S]^+^, abstracts the hydrogen at the C-5′ position followed by cyclization to form a C-7′ radical intermediate. The substrate based radical intermediate continually abstracts a hydrogen atom from the protein to give OAP. Additional detailed enzymatic mechanism studies including on which residue is the hydrogen atom source are currently being pursued in our laboratory.

### PolH and PolJ are capable of being utilized as potential probes for the discovery of new natural products

Although the OA moiety is a common structure shared by several high-carbon sugar nucleoside natural products including polyoxin and nikkomycin, the puzzle of its biosynthesis remained unsolved for over half a century. With the functional roles of PolH and PolJ distinctly dissected in this study, we could utilize both of them as probes for the target-directed genome mining of potential novel OA- or AHA-containing compounds with antifungal bioactivity, and we are gratified to have identified several gene clusters that also contain the conserved 6 genes including *polH* and *polJ*, suggesting that the OA, as an unusual building block for polyoxin, is diversely distributed in nature (Fig. 4, ESI and Table S3†). Furthermore, using *polH* and *polJ* as probes not only offers great potential for natural product discovery, in particular for those containing the OA moiety, but also provides new directions for future natural product development.

## Conclusions

**Scheme 3:**
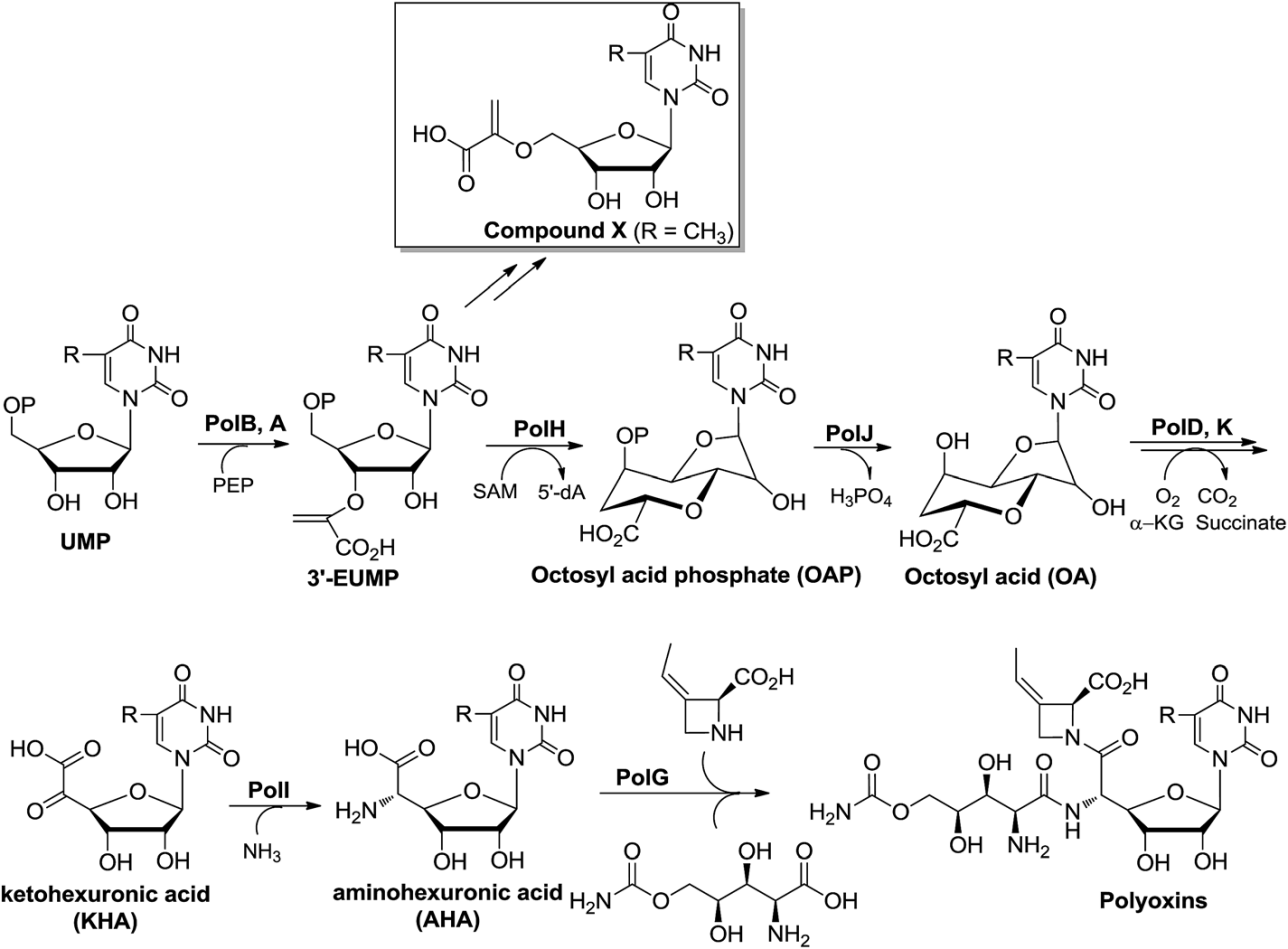
The proposed biosynthetic pathway for polyoxin. R = H, CH_3_, CH_2_OH or COOH.

**Fig. 5:**
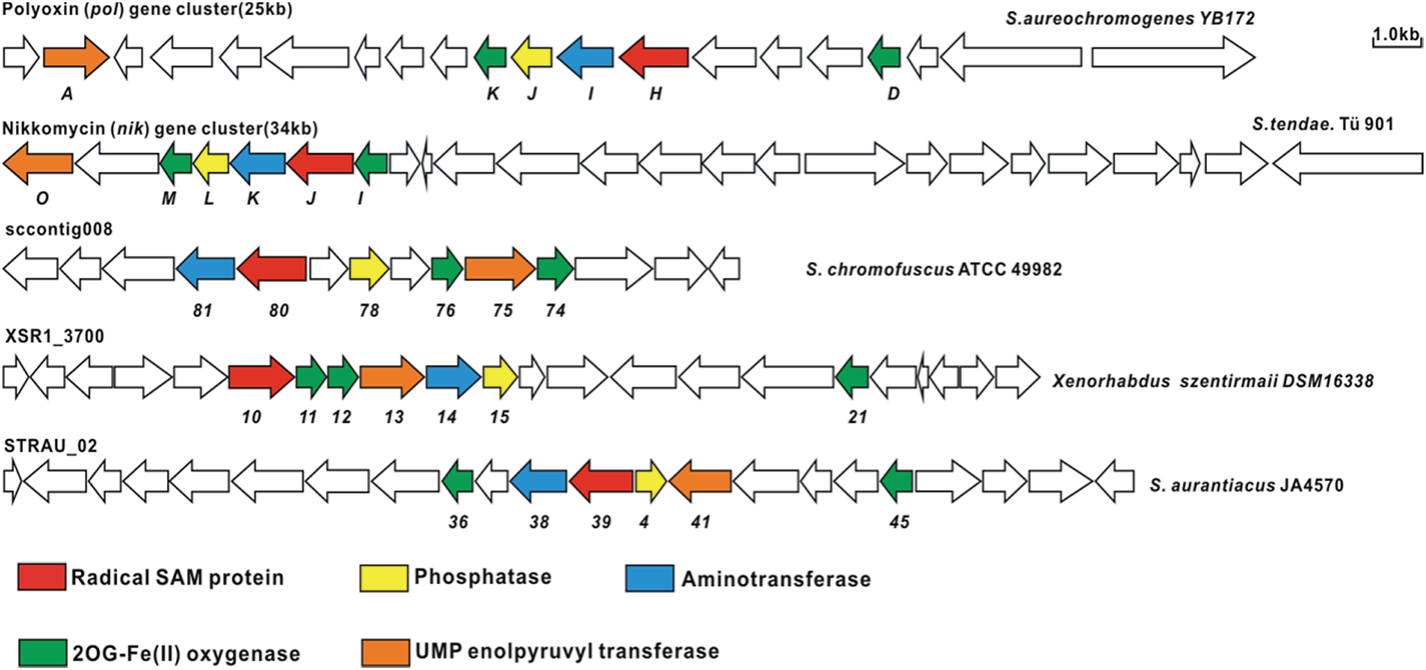
Target-directed genome mining of the potential nucleoside antibiotics containing AHA using PolH and PolJ as probes. The genome sequence data was obtained from GenBank, and the related ORFs associated with aminohexuronic acid (uracil polyoxin C) are linked together and probably encode potential nucleoside antibiotics related to polyoxin and nikkomycin.

In summary, we have dissected the initial two crucial enzymatic steps by PolH and PolJ leading to OA biosynthesis by a combination of *in vivo* and *in vitro* investigations. A revised biosynthetic pathway for polyoxin was proposed (Scheme 3), and the second key intermediate, AHA, is most likely generated from OA with C6′–C7′ bond cleavage to give ketohexuronic acid (KHA) by two putative α-KG dependent dioxygenases, PolD and PolK, followed by a PLP dependent aminotransferase, PolI, whose characterized homolog is NikK.^18^ Our studies revealed that construction of the OA backbone highlights a free radical strategy to first form OAP followed by dephosphorylation which agrees well with the labelling feeding experiments. More importantly, we unveiled several potential gene clusters coding for OA- or AHA-containing high-carbon sugar nucleoside antibiotics biosynthesized from diverse strains, by target-directed genome mining using PolH and PolJ as probes (Fig. 5, ESI and Table S3†). We anticipate that the deciphering of OA biosynthetic puzzles will contribute to polyoxin pathway engineering, and also enrich the toolbox for novel enzymatic mechanisms.
