# Supplementary material for: Construction of an octosyl acid backbone catalyzed by a radical S-adenosylmethionine enzyme and a phosphatase in the biosynthesis of high-carbon sugar nucleoside antibiotics
Source: Chem Sci. 2016 Aug 19;8(1):444–51. doi: 10.1039/c6sc01826b (PMC5365060; doi:10.1039/c6sc01826b)
Supplement: Supplementary file 1 [file SC-008-C6SC01826B-s001.pdf]

Supporting Information for

Construction of the Octosyl Acid Backbone Catalyzed by a Radical S-Adenosylmethionine Enzyme and a Phosphatase in the Biosynthesis of High-carbon Sugar Nucleoside Antibiotics

Nisha He,[†,‡,§] Pan Wu,[†,§] Yongxing Lei,[‡] Baofu Xu,[‡] Xiaochen Zhu,[‡] Gudan Xu,[†] Yaojie Gao,[†] Jianzhao Qi,[†] Zixin Deng,[†] Gongli Tang,[#] Wenqing Chen,*[,†] and Youli Xiao*[,‡]

[†]Key Laboratory of Combinatorial Biosynthesis and Drug Discovery, Ministry of Education, and School of Pharmaceutical Sciences, Wuhan University, Wuhan 430071, China

[‡]CAS Key Laboratory of Synthetic Biology, CAS Center for Excellence in Molecular Plant Sciences, Institute of Plant Physiology and Ecology, Shanghai Institutes for Biological Sciences, Chinese Academy of Sciences, 300 Fenglin Road, Shanghai 200032, China

[#]State Key Laboratory of Bio-organic and Natural Products Chemistry, Shanghai Institute of Organic Chemistry, Chinese Academy of Sciences, 345 Lingling Road, Shanghai 200032, China

[§]These authors contributed equally.

***Corresponding Author:** ylxiao@sibs.ac.cn, wqchen@whu.edu.cn

Table of Contents

1. Supplementary Figures

Figure S1. Examples of nucleoside antibiotics containing high-carbon (>6 C's) sugar scaffold.

Figure S2. In-frame deletion of *polJ* or *polH* on pJTU4774.

Figure S3. Bioassay and chemical analysis of the metabolites accumulated by *ΔpolH* or *ΔpolJ* mutants.

Figure S4. ^{1}H NMR of compound X in D_2O (500 MHz).

Figure S5. ^{13}C NMR of compound X in D_2O (125 MHz).

Figure S6. DEPT NMR of compound X in D_2O.

Figure S7. ^{1}H-^{1}H COSY of compound X in D_2O.

Figure S8. HMQC of compound X in D_2O.

Figure S9. HMBC of compound X in D_2O.

Figure S10. Bioinformatic analysis of PolJ with its homologs.

Figure S11. Bioinformatic analysis of PolH with its homologs.

Figure S12. Overexpression, purification, SDS-PAGE and ESI-MS/MS analysis of purified PolJ.

Figure S13. Purification, reconstitution, SDS-PAGE and UV-Vis analysis of the purified PolH.

Figure S14. EPR characterization of as-isolated and reconstituted PolH.

Figure S15. Analysis of NikO and LC-MS confirmation of its catalyzed-product, 3'-EUMP.

Figure S16. LC-MS/MS analysis of the reaction of 3'-EUMP catalyzed by PolJ.

Figure S17. LC-MS/MS analysis of the reaction of 3'-EUMP catalyzed by PolJ-PolH.

Figure S18. PolH activity assay.

Figure S19. ^{1}H-, ^{13}C-, ^{31}P-, and DEPT NMRs analysis of purified product from the reaction of 3'-EUMP catalyzed by PolH with the contamination of glycerol.

Figure S20. ^{1}H-NMR and ^{13}C-NMR of the glycerol in D_2O solvent.

Figure S21: ^{1}H-^{1}H COSY of the OAP with glycerol contamination.

Figure S22: HMQC of the OAP with glycerol contamination.

Figure S23: HMBC of the OAP with glycerol contamination.

Figure S24. LC-MS/MS analysis of the reaction of OAP catalyzed by PolJ.

Figure S25: The time course of PolH activity assay with the stoichiometric production of OAP and 5'-dA.

Figure S26: The PolH reaction in the 60% of D_2O buffer solution.

2. Supplementary Scheme

Scheme S1. PolJ and PolH reaction with 3'-EUMP as substrate.

3. Supplementary Tables

Table S1. Strains, plasmids, and cosmids used in this study.

Table S2. PCR primers used in this study.

Table S3. Comparative analysis of the target *pol* genes and their homologs involved in potential AHA biosynthetic pathway.

4. Methods and Experimental section

Method S1. Materials, instruments and general methods.

Method S2. In frame deletion of *polH* or *polJ* in cosmid pJTU4774 by PCR-targeting technology.

Method S3. Cultivation and metabolites analysis of the *S. aureochromogenes* and its derivatives .

Method S4. Preparation of the NikO-catalyzed product, 3'-EUMP.

Method S5. Cloning of PolH and PolJ in *E. coli.*

Method S6. Over-expression and purification of PolJ.

Method S7. PolJ activity assays.

Method S8. Purification and reconstitution of PolH protein.

Method S9. PolH activity assays.

5. Supplementary References

Reference S1. Supplementary References.

Figure S1. Examples of nucleoside antibiotics containing high-carbon (> 6 C's) sugar scaffold.

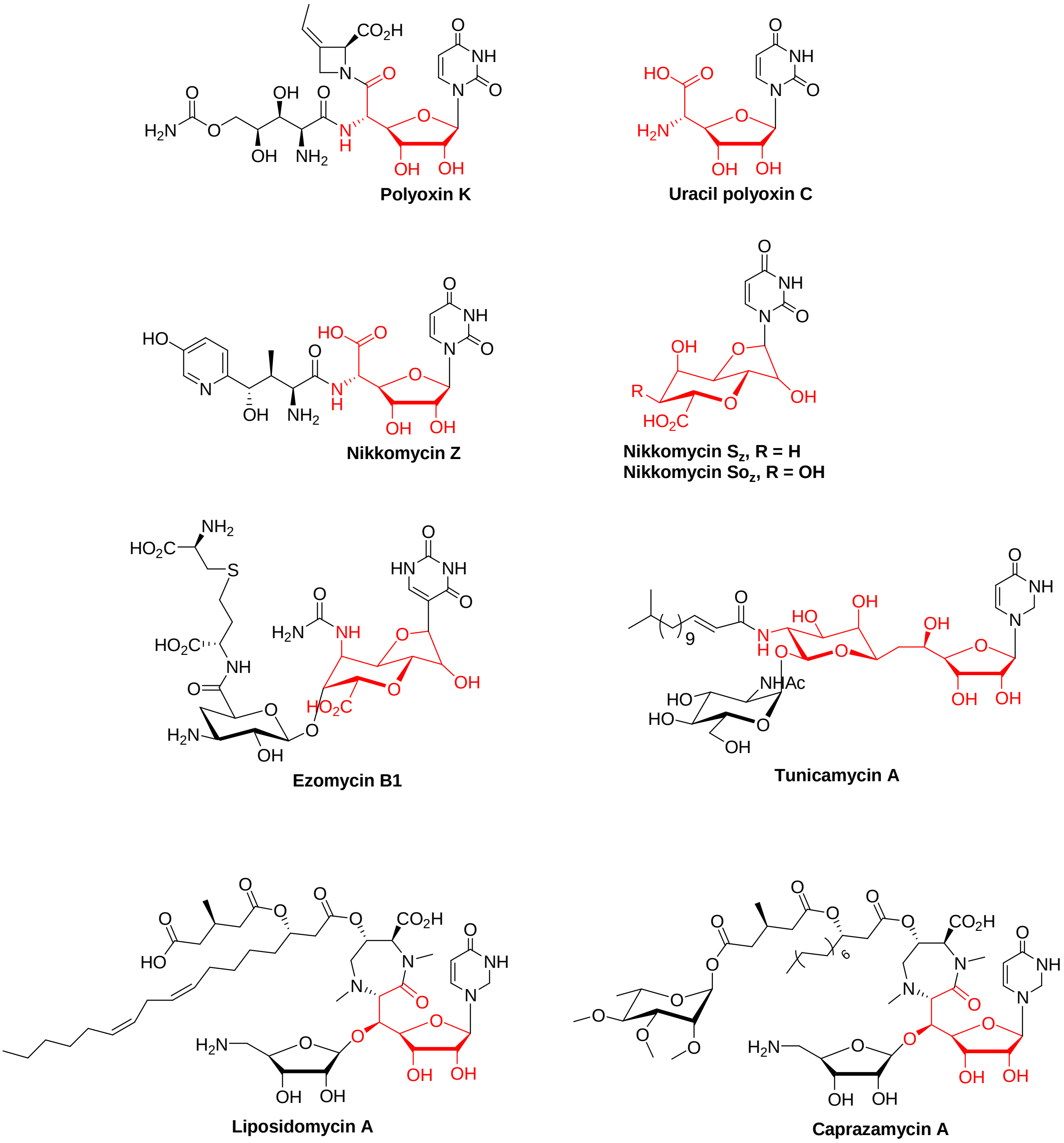

Figure S2. In-frame deletion of *polJ* or *polH* on pJTU4774.

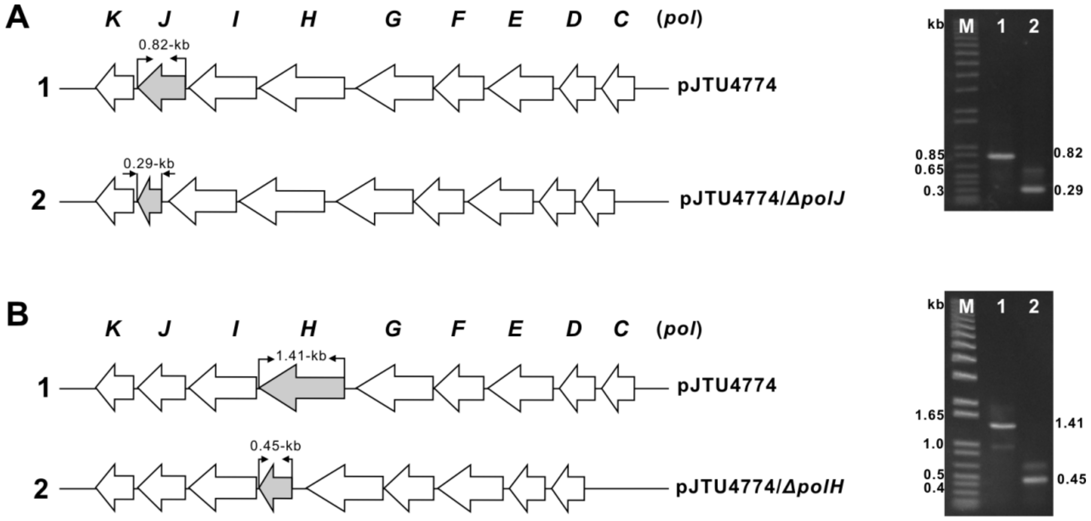

(A) Schematic map for the in-frame deletion of *polJ* on pJTU4774 (left) and the PCR confirmation (right).

(B) Schematic map for the in-frame deletion of *polH* on pJTU4774 (left) and the PCR confirmation (right). M, 1 kb plus ladder; 1, WT; 2 and 3, samples corresponding to the numbered gene clusters to the left.

Figure S3. Bioassay and chemical analysis of the metabolites accumulated by *ΔpolH* or *ΔpolJ* mutants.

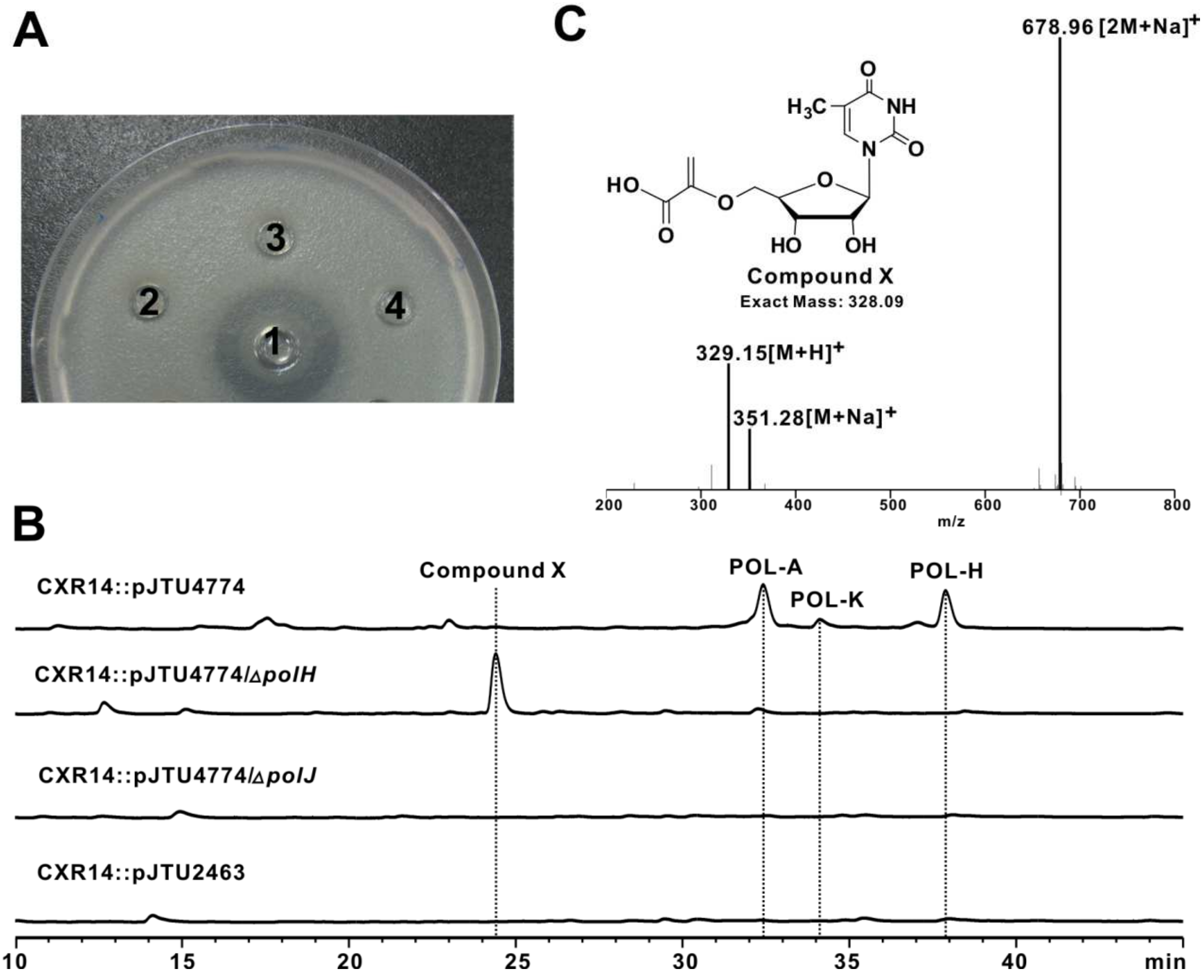

(A) Bioassay of the metabolites produced by related strains. **1**, Metabolites produced by CXR14::pJTU4774; **2**, Metabolites produced by CXR14::pJTU774/*ΔpolH*; **3**, Metabolites produced by CXR14::pJTU774/*ΔpolJ*; **4**, Metabolites produced by CXR14::pJTU2463 as negative control. *Trichosporon cutaneum* 3071 was selected as indicator fungi for the bioassay of polyoxins and related metabolites.

(B) HPLC analysis of the metabolites produced by related strains, CXR14 was the strain with the entire polyoxin gene cluster deleted, and this strain is facilitated for the genetic analysis of the target genes involved in polyoxin biosynthesis.

(C) LC-MS analysis of the compound **X**. This compound indicates a $[M+H]^+$ ion at *m/z* 329.15 whose structure was further solved by comprehensive NMR analysis.

Figure S4. ^{1}**H NMR of compound X in D_2O (500 MHz).**

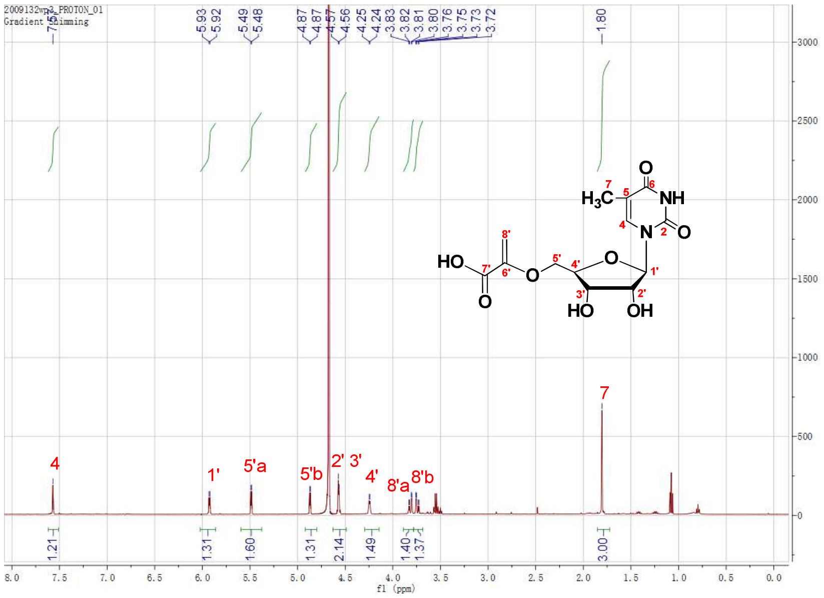

^{1}**H NMR** (500 MHz, D_2O) δ 7.57 (1H, s), 5.93 (1H, d, J = 5.0 Hz) , 5.49 (1H, d, J = 5.0 Hz), 4.87 (1H, s), 4.57 (2H, d, J = 5.0 Hz), 4.24 (1H, d, J = 5.0 Hz), 3.83 (1H, dd, J = 5.0, 5.0 Hz), 3.76 (1H, dd, J = 5.0, 5.0 Hz), 1.80 (3H, s).

Figure S5. ^{13}**C NMR of compound X in D_2O (125 MHz).**

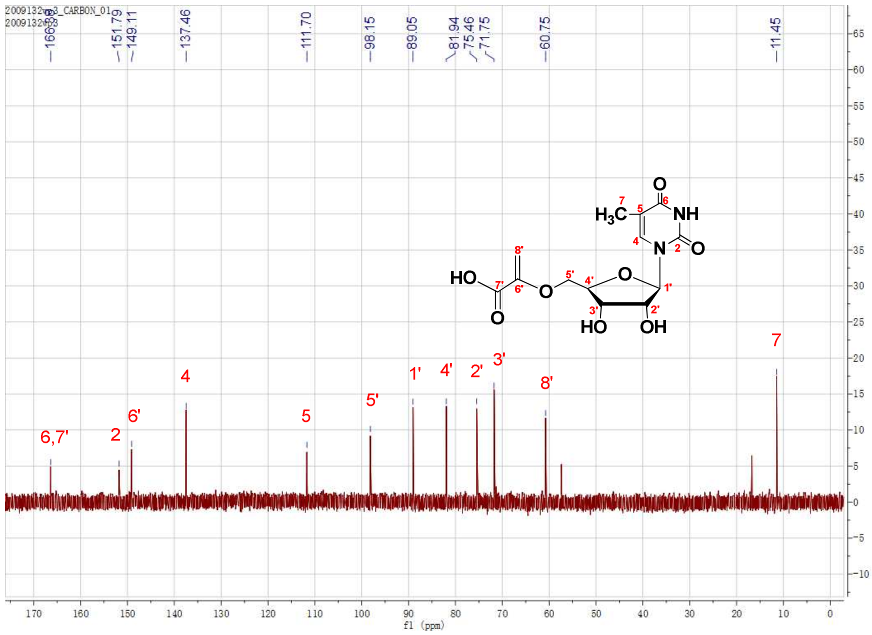

^{13}**C NMR** (125 MHz, D_2O) δ 166.40, 166.38, 151.79, 149.11, 137.46, 111.70, 98.15, 89.05, 81.94, 75.46, 71.75, 60.75, 11.45.

Figure S6. DEPT NMR of compound X in D_2O.

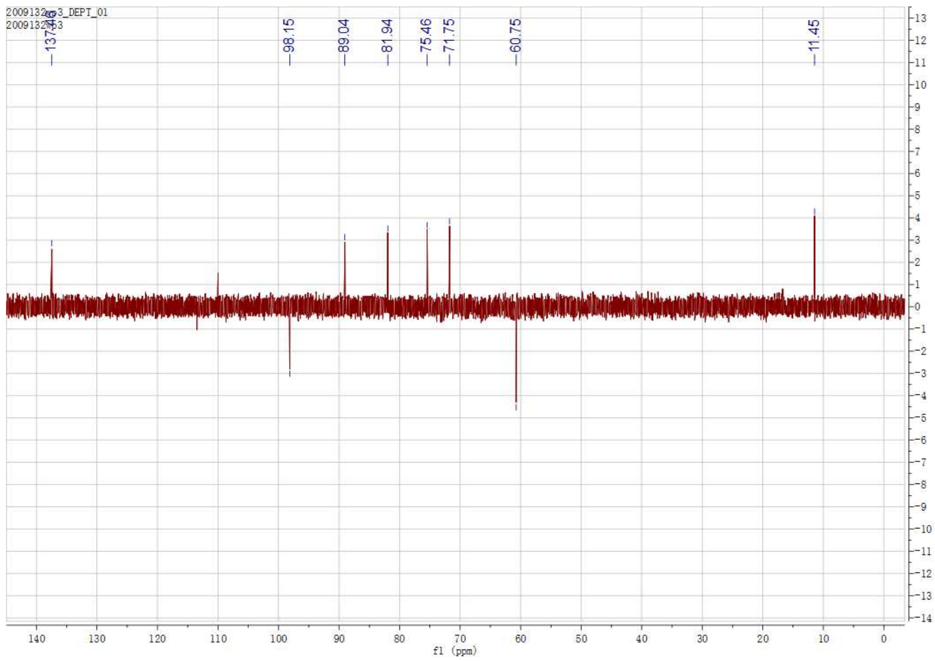

Figure S7. ^{1}H-^{1}H COSY of compound X in D_2O.

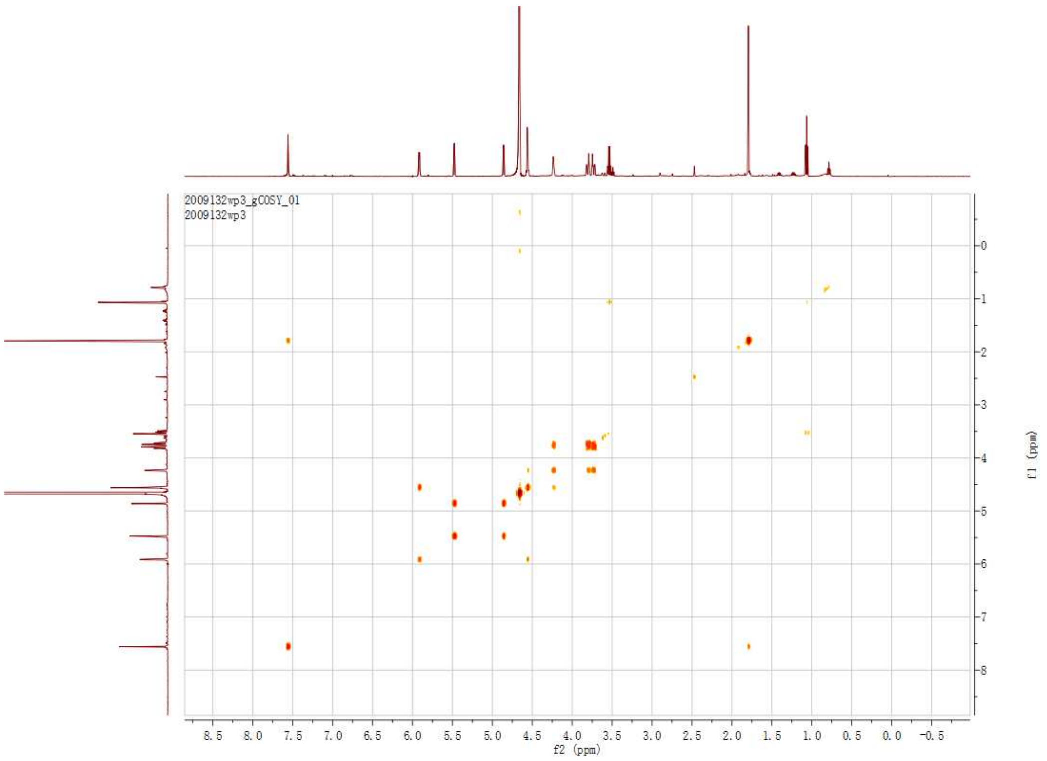

Figure S8. HMQC of compound X in D_2O.

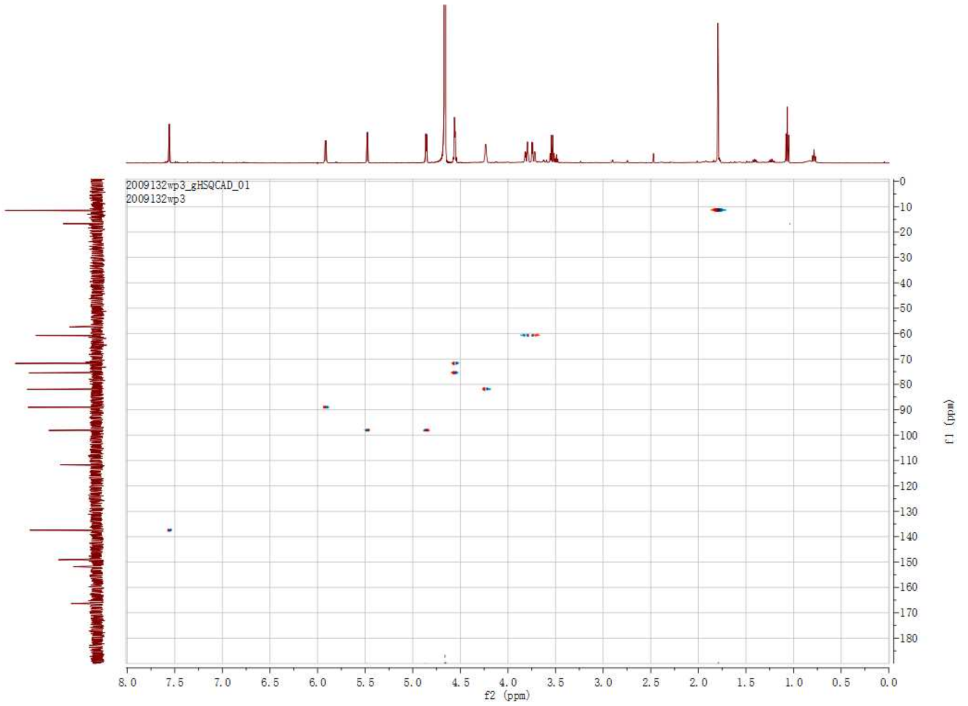

Figure S9. HMBC of compound X in D_2O.

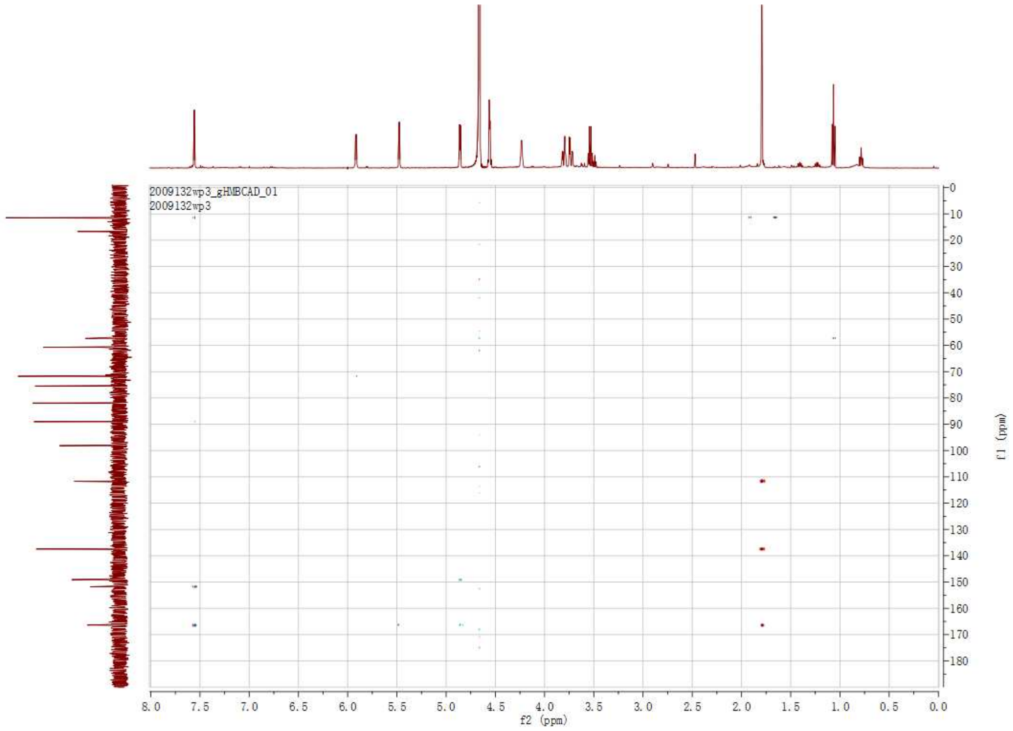

Figure S10. Bioinformatic analysis of PolJ with its homologs.

Proteins include PolJ (*S. aureochromogenes* YB172), NikL (*S. tendae* Tü901), sccontig008-78 *S. chromofuscus* ATCC49982, STRAU_0240 (*S. aurantiacus* JA4570), and XSR1_370020 *(Xenorhabdus szentirmaii* DSM16338). The sequences were aligned with ClustalW and merged with ESpript online program.

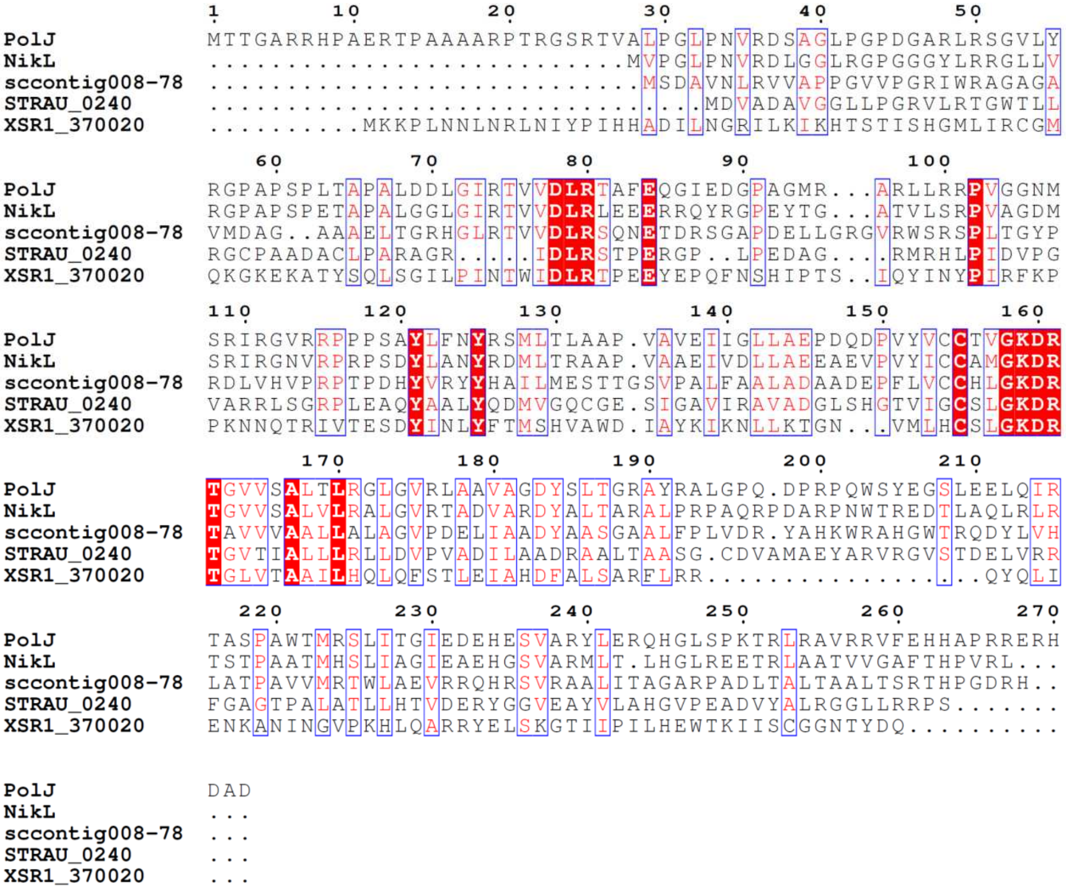

Figure S11. Bioinformatic analysis of PolH with its homogs

Proteins include PolH (*S. aureochromogenes* YB172), NikJ (*S. tendae* Tü901), sccontig008-80 *S. chromofuscus* ATCC49982, STRAU_0246 (*S. aurantiacus* JA4570), and XSR1_370015 (*Xenorhabdus szentirmaii* DSM16338). The sequences were aligned with ClustalW and merged with ESpript online program.

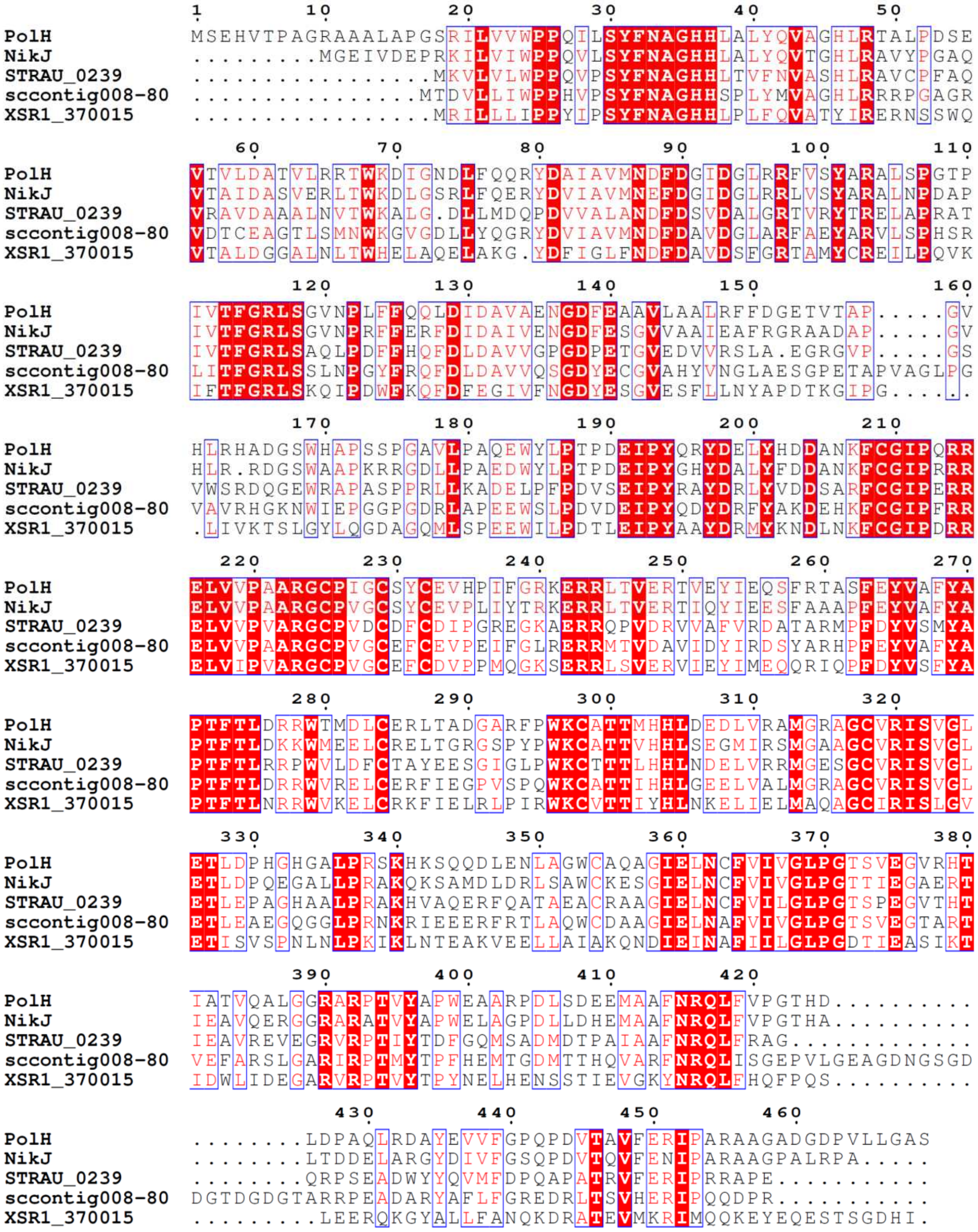

Figure S12. Overexpression, purification, SDS-PAGE and ESI-MS/MS analysis of purified PolJ.

(A) Overexpression and purification of PolJ protein. A culture of *E. coli* BL21(DE3) cells harboring the pJTU2864 construct were grown in the LB medium containing 50 μg/mL of kanamycin at 37 °C with shaking (200 rpm) until the OD_{600} reached ~0.5. Protein expression was then induced by addition of isopropyl β-D-1-thiogalactopyranoside (IPTG) to a final concentration of 0.5 mM, and the cells were allowed to grow at 18 °C and 200 rpm for an additional 18 h. The cells were harvested by centrifugation at 4500 × g for 15 min. All purification steps were carried out at 4 °C using Ni-NTA resin and the PolJ protein were washed and eluted by 20-250 mM imidazole in 25 mM Tris-HCl, 150 mM NaCl buffer (pH 8.0). The collected protein solution was dialyzed against 25 mM Tris-HCl, 150 mM NaCl buffer (pH 8.0) for three times. The protein solution was then flash-frozen in liquid nitrogen and stored at -80 °C until use. Protein concentration was determined by the Bradford assay using bovine serum albumin as the standard.

(B) SDS-PAGE analysis of purified PolJ.

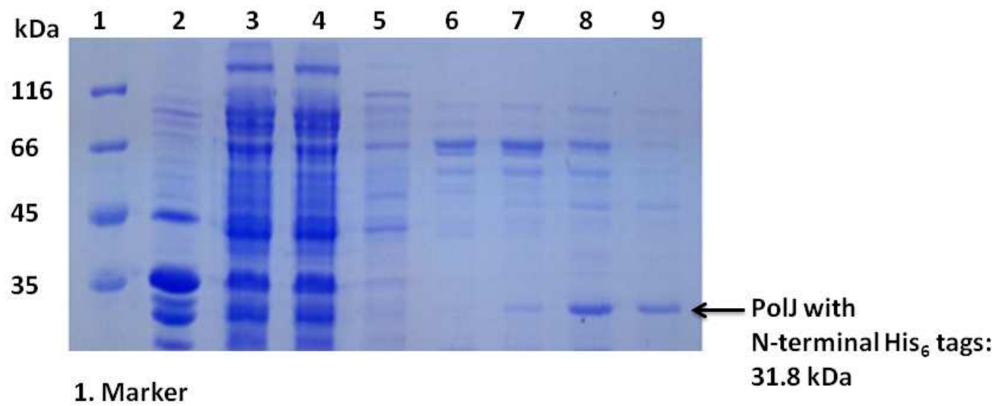

1. Marker
2. Precipitate of PolJ expressing *E. coli*
3. Supernatant of the homogenate of PolJ expressing *E. coli*
4. Flow-through from Ni column
5. Washing with washing buffer
6. Elution with 20 mM imidazole in elution buffer
7. Elution with 50 mM imidazole in elution buffer
8. Elution with 100 mM imidazole in elution buffer
9. Elution with 250 mM imidazole in elution buffe

(C) ESI-MS/MS analysis the unique peptides from the purified PolJ digested by Trypsin.

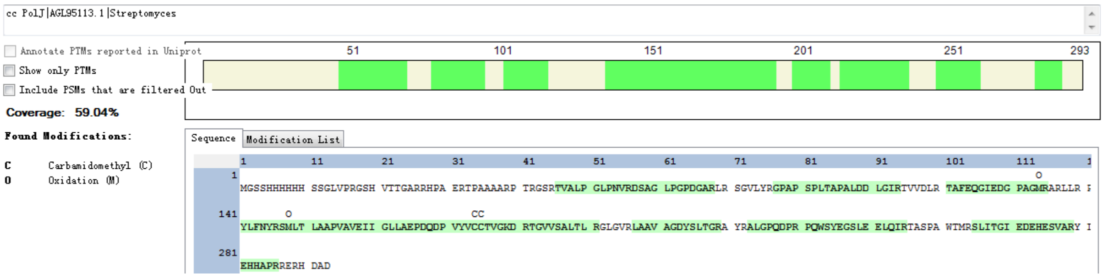

Figure S13. Purification, reconstitution, SDS-PAGE and UV-Vis analysis of the purified PolH.

(A) Purification and reconstitution of PolH protein. Following the similar procedure as the over-expression and purification of PolJ, PolH was purified anaerobically from the *E. coli* BL21(DE3)/plysE cells harboring the pJTU2877 construct in 25 mM Tris-HCl, 150 mM NaCl, 5% glycerol buffer (pH 8.0). The reconstitution was performed by incubation of as-isolated PolH with 6 equal excess mole amount of Na_2S, $Fe(NH_4)_2(SO_4)_2$ in presence of 5.0 mM DTT for 3 hr at 4 ^oC anaerobically.

(B) SDS-PAGE of the purification of PolH.

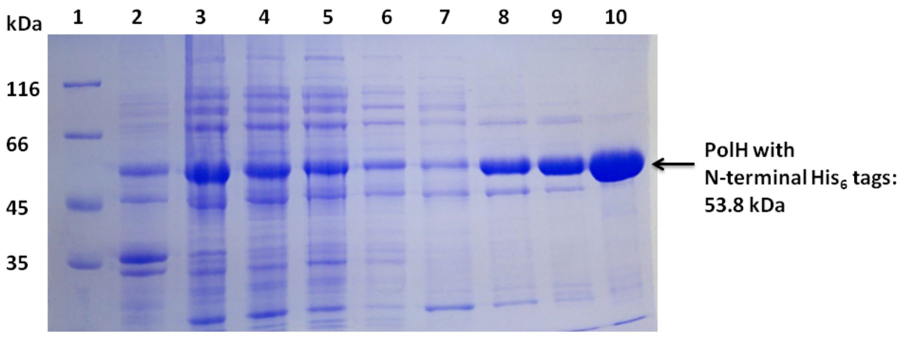

1. Marker
2. Precipitate of PolH expressing *E. coli*
3-4. Supernatant of the homogenate of PolH expressing *E. coli*
5. Flow-through from Ni column
6. Washing with washing buffer
7. Elution with 20 mM imidazole in elution buffer
8. Elution with 50 mM imidazole in elution buffer
9. Elution with 100 mM imidazole in elution buffer
10. Elution with 250 mM imidazole in elution buffer

(C) UV-Vis spectra of as-isolated, reconstituted and reduced PolH.

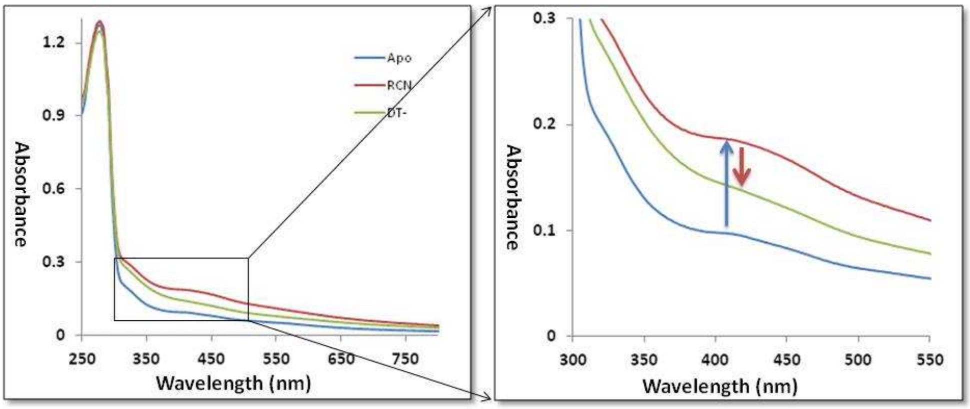

1. Apo: As-isolated PolH in anaerobic condition
2. RCN: Reconstituted PolH with Fe^{2+} and S^{2-} in presence of DTT
3. DT-: Partially reduced reconstituted PolH by diothionite

Figure S14. EPR characterization of as-isolated and reconstituted PolH.

(A)

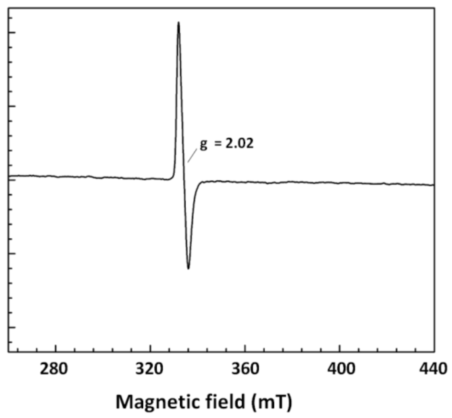

(B)

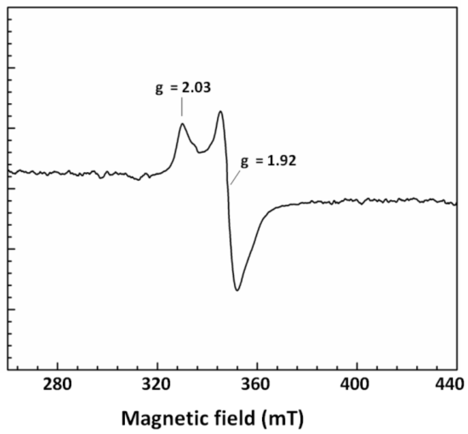

(C)

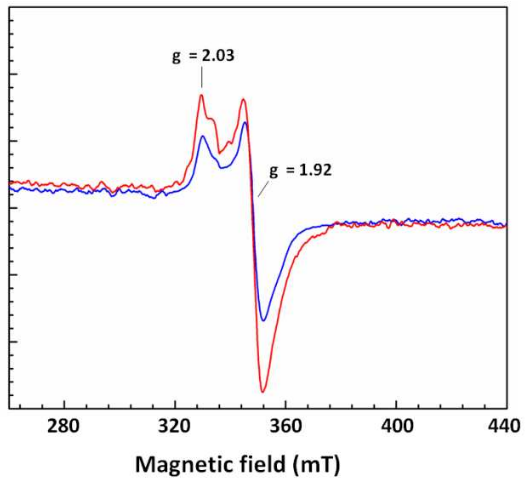

(A) EPR spectrum of as-isolated PolH (500 µM, in 25 mM Tris-HCl, 150 mM NaCl, 10% glycerol buffer, pH 8.0).

(B) EPR spectrum of as-isolated PolH reduced by sodium dithionite.

As-isolated PolH (500 µM, in 25 mM Tris-HCl, 150 mM NaCl, 10% glycerol buffer, pH 8.0) was incubated with 5.0 mM sodium dithionite anaerobically on ice for 5.0 minutes and frozen by liquid nitrogen for EPR recording.

(C) EPR spectra of reduced as-isolated (blue line) and reconstituted (red line) PolH.

The reduced as-isolated EPR sample (blue line) is same as above (B). The reduced reconstituted (red line) PolH (400 µM) was prepared with similar condition as above B and same as Fig. 2C.

Figure S15. Analysis of NikO and LC-MS confirmation of its catalyzed-product, 3’-EUMP.

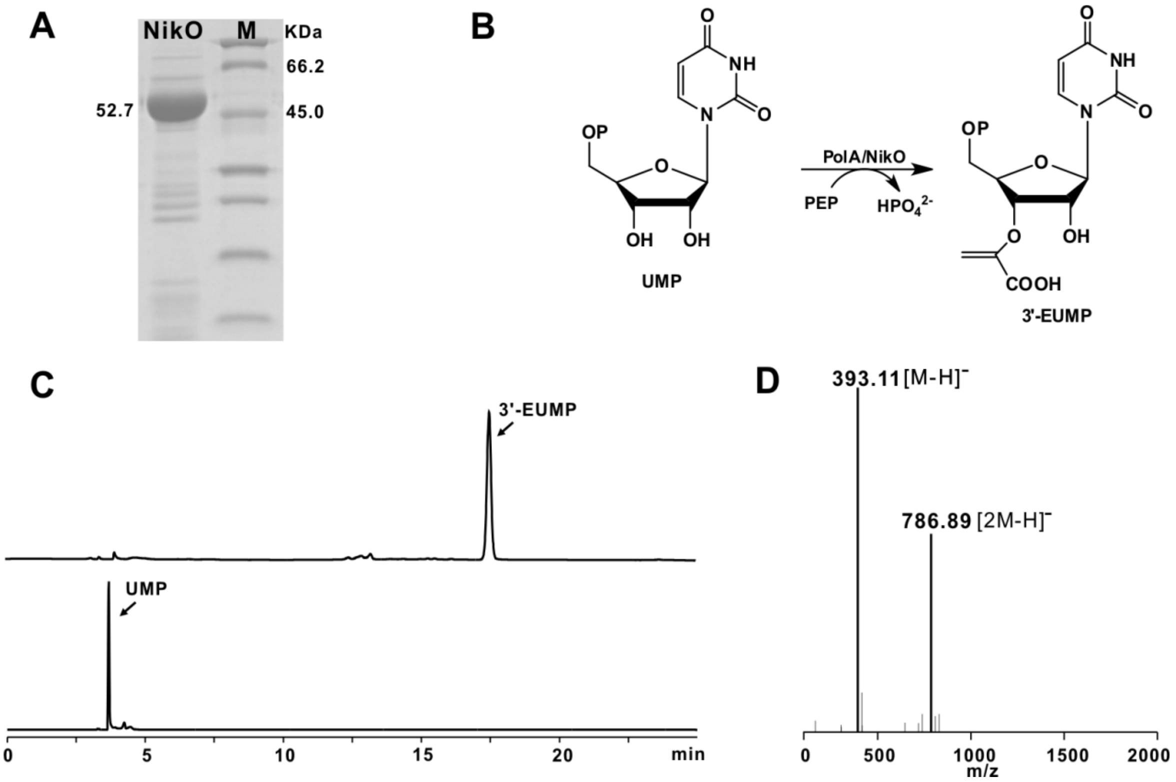

(A) SDS-PAGE analysis of the purified NikO.

(B) Schematic map of the catalyzed reaction by NikO or it's homolog, PolA.

(C) HPLC analysis of the UMP reaction catalyzed by NikO. Top, NikO reaction; bottom, negative control without NikO added.

(D) LC-MS confirmation of the NikO-catalyzed product, 3’-EUMP, which indicated a [M-H]$^-$ ion at *m/z* 393.11, fully corresponding to its theoretical mass.

Figure S16. LC-MS/MS analysis of the reaction of 3'-EUMP catalyzed by PolJ.

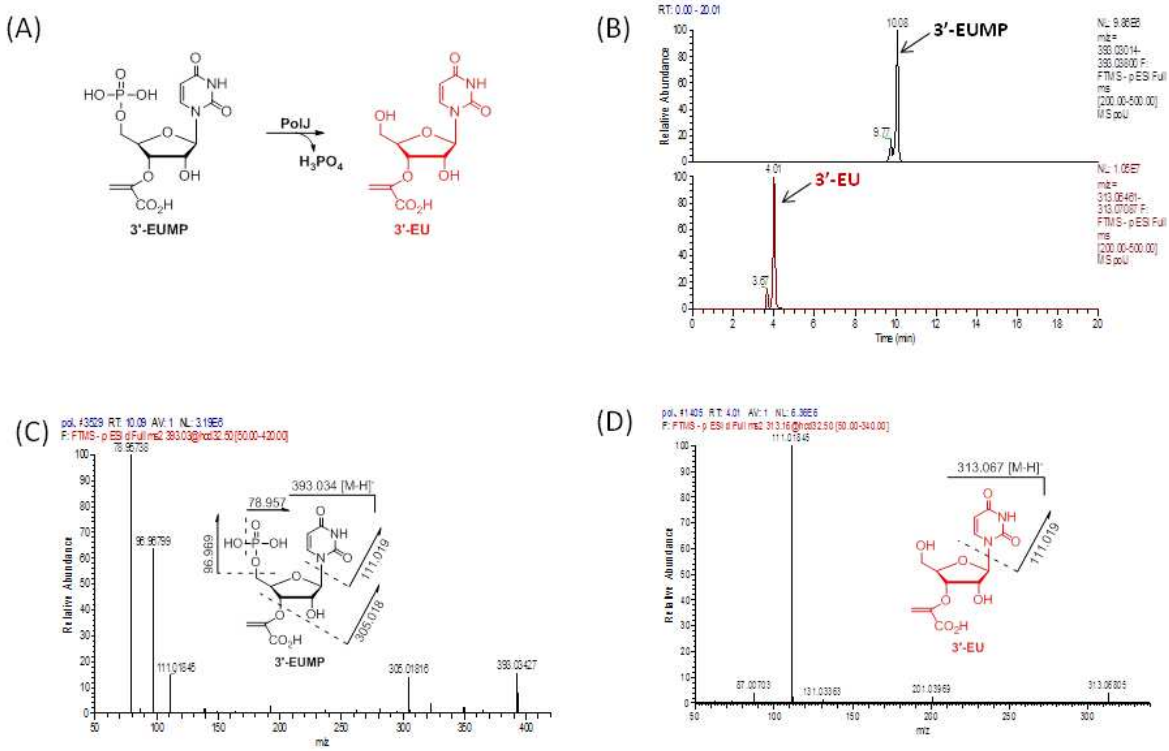

(A) PolJ reaction using 3'-EUMP as substrate. The purified N-His$_6$-PoHJ protein was incubated with 3'-EUMP (1.0 mM) and $MgCl_2$ (1.0 mM) in 50 mM Tris-HCl bufer (pH 8.0) at 25°C for 8 hr.

(B) LC-MS/MS analysis of reaction mixture.

(C) Comparative tandem MS analysis of the substrate 3'-EUMP (from left to right: 78.967, 96.967, 111.018, 305.018, 393.034)**.**

(D) Comparative tandem MS analysis of the product 3'-EU (from left to right: 87.007, 111.018, 201.039, 313.068)**.**

Figure S17. LC-MS/MS analysis of the reaction of 3'-EUMP catalyzed by PolJ-PolH.

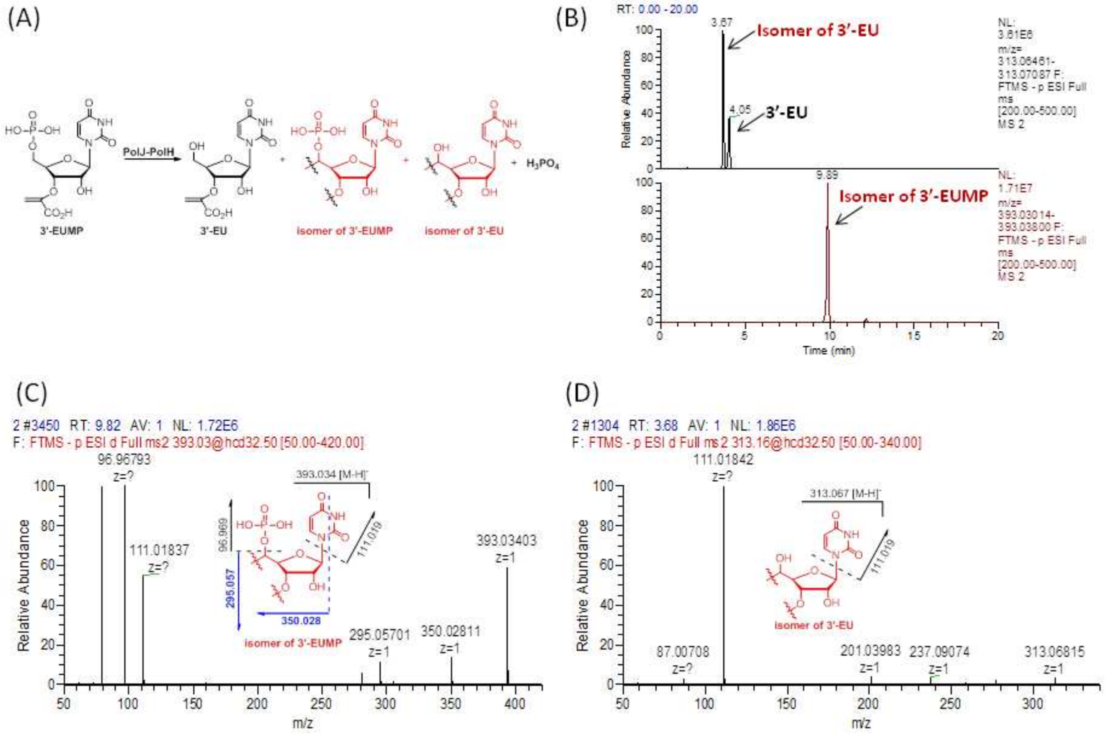

(A) PolJ-PolH reaction using 3'-EUMP as substrate. The purified N-His$_6$-PoHJ and anaerobically reconstituted PolH proteins was incubated anaerobically with 3'-EUMP (1.0 mM), $MgCl_2$ (1.0 mM), SAM (1.0 mM), and sodium dithionite (2.0 mM) in 50 mM Tris-HCl bufer (pH 8.0) at 25°C for 8 hr.

(B) LC-MS/MS analysis of reaction mixture.

(C) Comparative tandem MS analysis of the product isomer of 3'-EUMP (from left to right: 96.967, 111.018, 295.057, 350.028, 393.034)**.**

(D) Comparative tandem MS analysis of the product isomer of 3'-EU (from left to right: 87.007, 111.018, 201.039, 237.090, 313.068)**.**

Figure S18. PolH activity assay.

(A) HPLC analysis of the PolH reaction with 3'-EUMP as substrate.

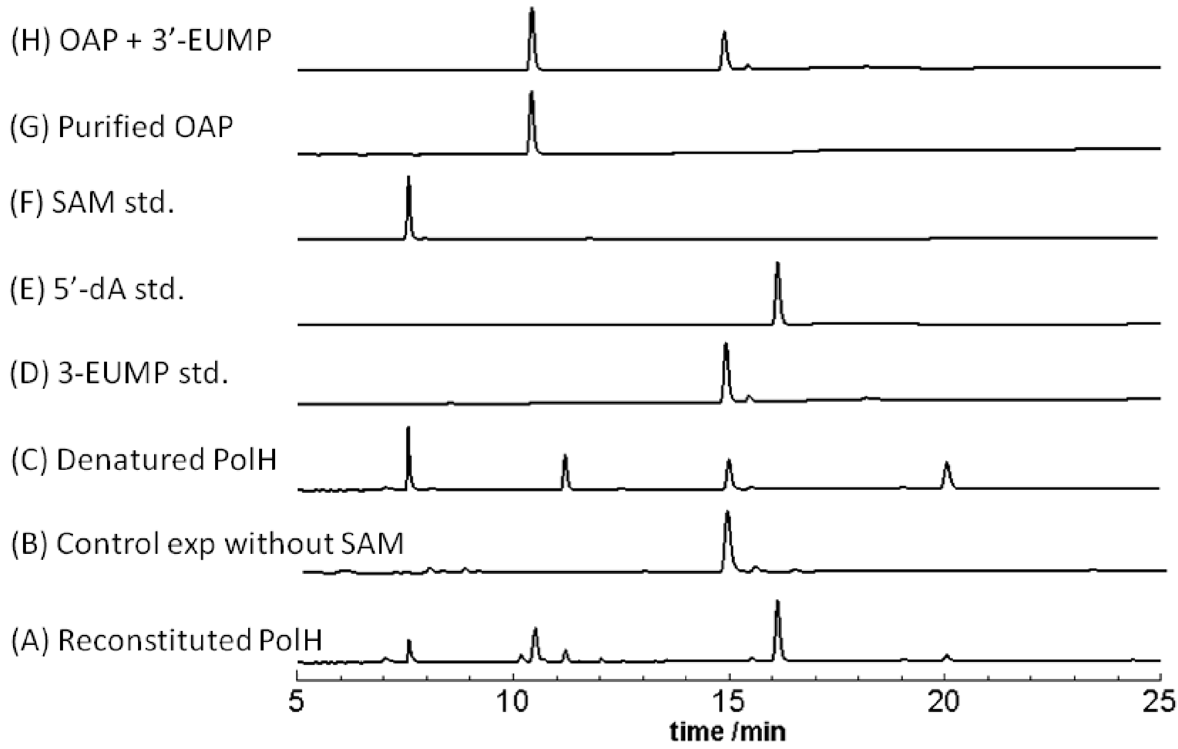

(A) Reconstituted PolH (20 µM) in presence of 3'-EUMP (0.5 mM), SAM (1.0 mM) and dithionite (2.0 mM) at 37 °C for 2.0 hr anaerobically; (B) the control assay without SAM; (C) the control assay with boiled denatured PolH; (D) 3'-EUMP standard; (E) 5'-dA standard; (F) SAM standard; (G) purified PolH reaction product (OAP); (H) co-injection of OAP with 3'-EUMP. **HPLC condition:** The samples were injected into a HPLC (Agilent 1260) equipped with a phenomenex Luna C18 column (5 µm, 4.6 x 250 mm) and eluted with 5%-30% MeOH (0.15% TFA) for 10 min, 30-50% MeOH (0.15% TFA) in 10-25 min with water as secondary mobile phase at a flow rate of 0.5 mL/min at 35 °C, monitored at 260 nm.

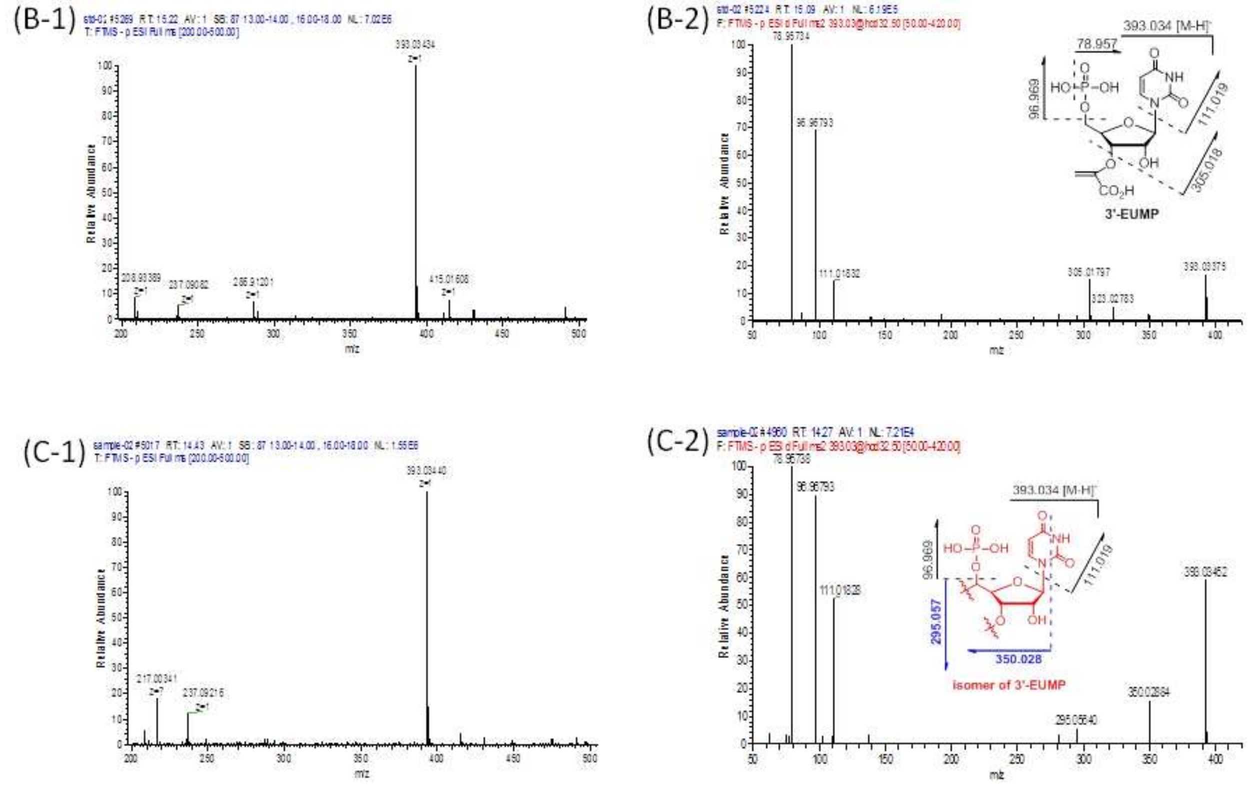

(B) LC-MS and ESI-MS/MS analysis of the substrate of 3'-EUMP catalyzed by PolH (B-1: 393.034; B-2 (from left to right): 78.957, 96.967, 111.018, 305.017, 393.033)**.**

(C) LC-MS and ESI-MS/MS analysis of the product isomer of 3'-EUMP catalyzed by PolH (C-1: 393.034; C-2 (from left to right): 78.957, 96.967, 111.018, 295.056, 350.029,393.034)**.**

Figure S19. ^{1}H-, ^{13}C-, ^{31}P-, and DEPT NMRs analysis of purified product from the reaction of 3'-EUMP catalyzed by PolH with the contamination of glycerol.

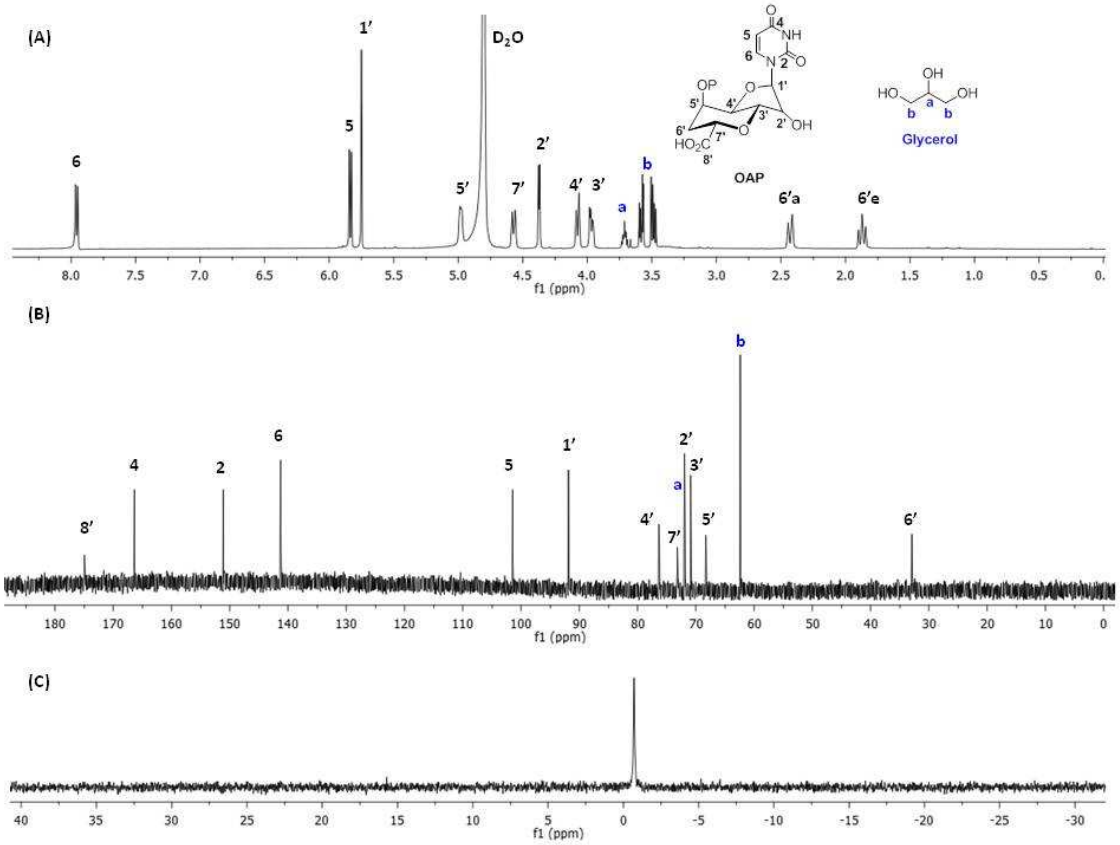

(A) ^{1}**H NMR** (500 MHz, D_2O) δ 7.96 (1H, d, *J* = 5.0 Hz), 5.84 (1H, d, *J* = 5.0 Hz) , 5.75 (1H, s), 4.98 (1H, d, *J* = 5.0 Hz), 4.57 (1H, d, *J* = 10.0 Hz), 4.38 (1H, d, *J* = 5.0 Hz), 4.08 (1H, d, *J* = 15.0 Hz), 3.96 (1H, dd, *J* = 10.0, 5.0 Hz), 2.42 (1H, d, *J* = 15.0 Hz), 1.87 (1H, t, *J* = 15.0 Hz). The signals of 3.70-3.72 (1H, m), 3.58 (2H, dd, *J* = 15.0, 5.0 Hz), and 3.48 (2H, dd, *J* = 10.0, 5.0 Hz) were from the glycerol contamination.

(B) ^{13}**C NMR** (125 MHz, D_2O) δ 174.95, 166.39, 151.13, 141.29, 101.47, 91.88, 76.36, 71.95, 70.89, 68.28, 32.93. The signals of 71.98 ppm and 62.39 ppm were from the glycerol contamination.

(C) ^{31}**P NMR** (202 MHz, D_2O) δ -0.71.

(D) **DEPT NMR.**

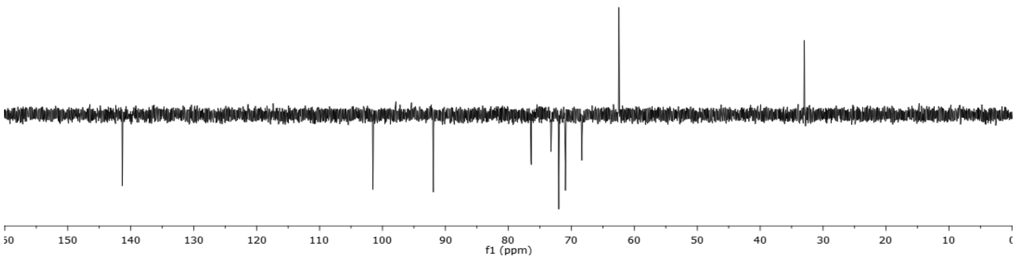

Figure S20. ^{1}H-NMR and ^{13}C-NMR of the glycerol in D_2O solvent.

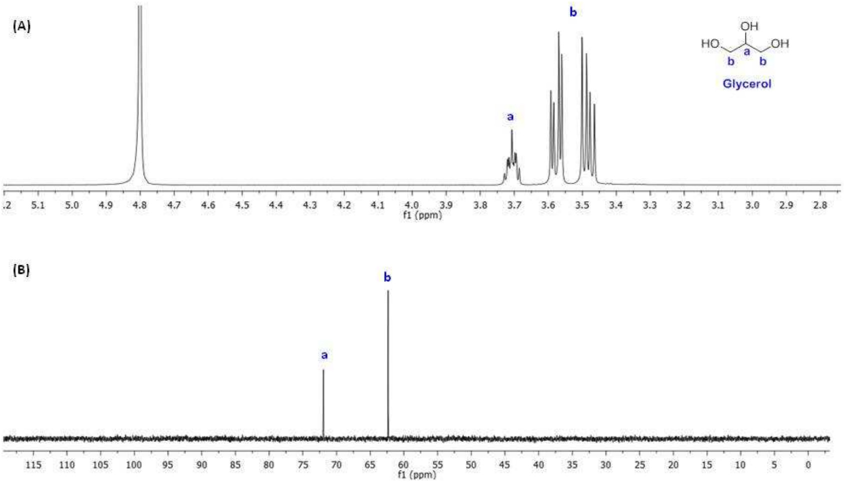

(A) ^{1}**H NMR** (500 MHz, D_2O) δ 3.70-3.72 (1H, m), 3.58 (2H, dd, *J* = 15.0, 5.0 Hz), 3.48 (2H, dd, *J* = 10.0, 5.0 Hz).

(B) ^{13}**C NMR** (125 MHz, D_2O) δ 71.94, 62.33.

Figure S21: ^{1}H-^{1}H COSY of the OAP with glycerol contamination.

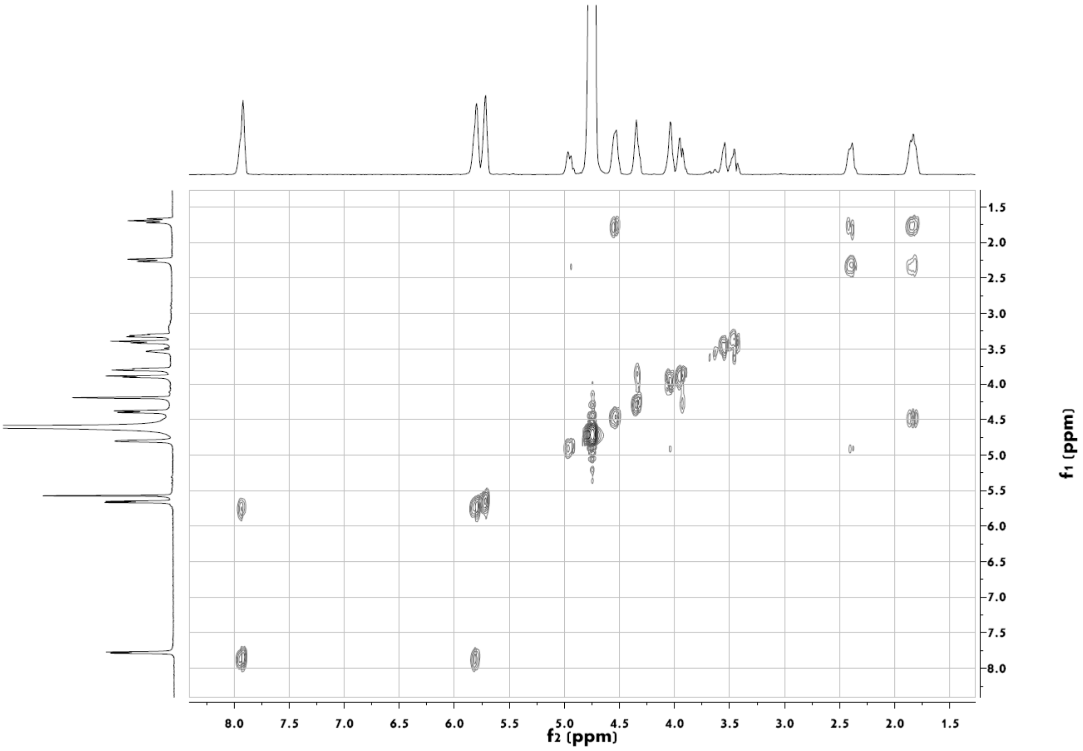

Figure S22: HMQC of the OAP with glycerol contamination.

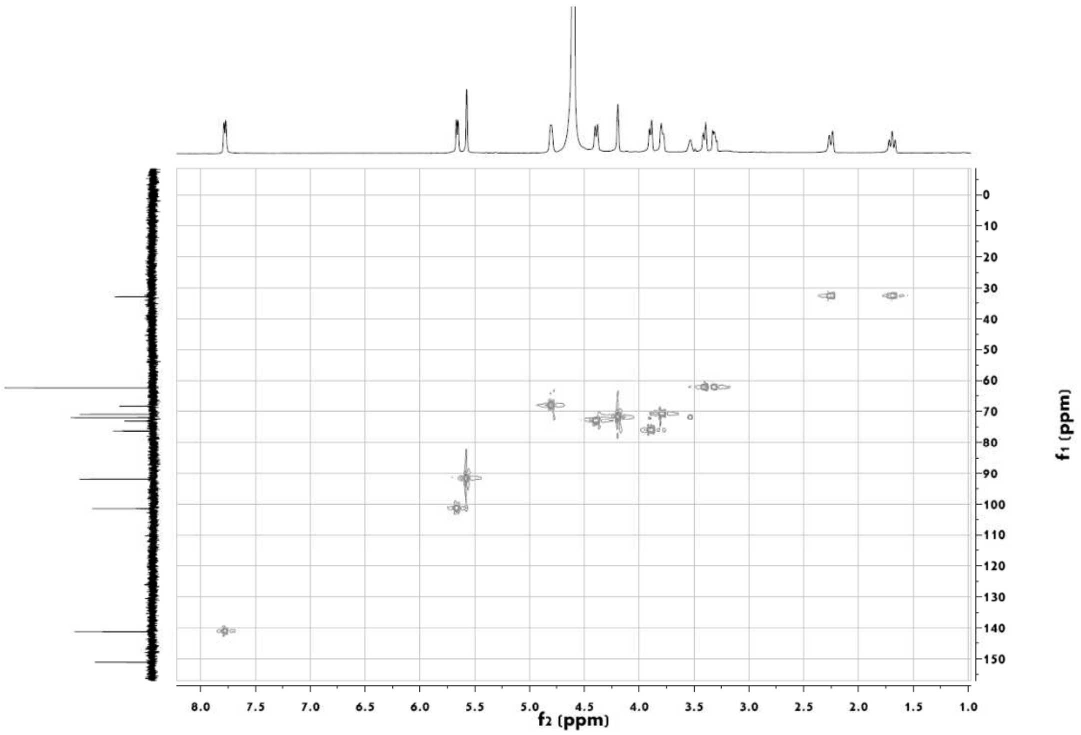

Figure S23: HMBC of the OAP with glycerol contamination.

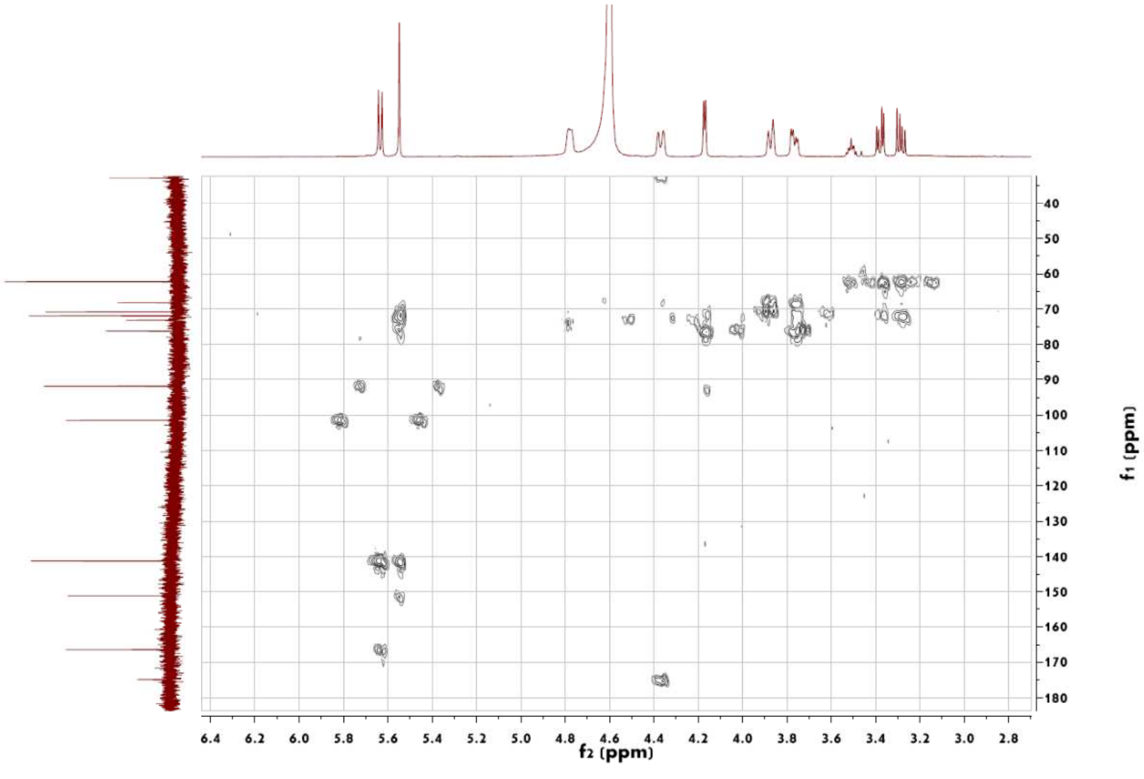

Figure S24. LC-MS/MS analysis of the reaction of OAP catalyzed by PolJ.

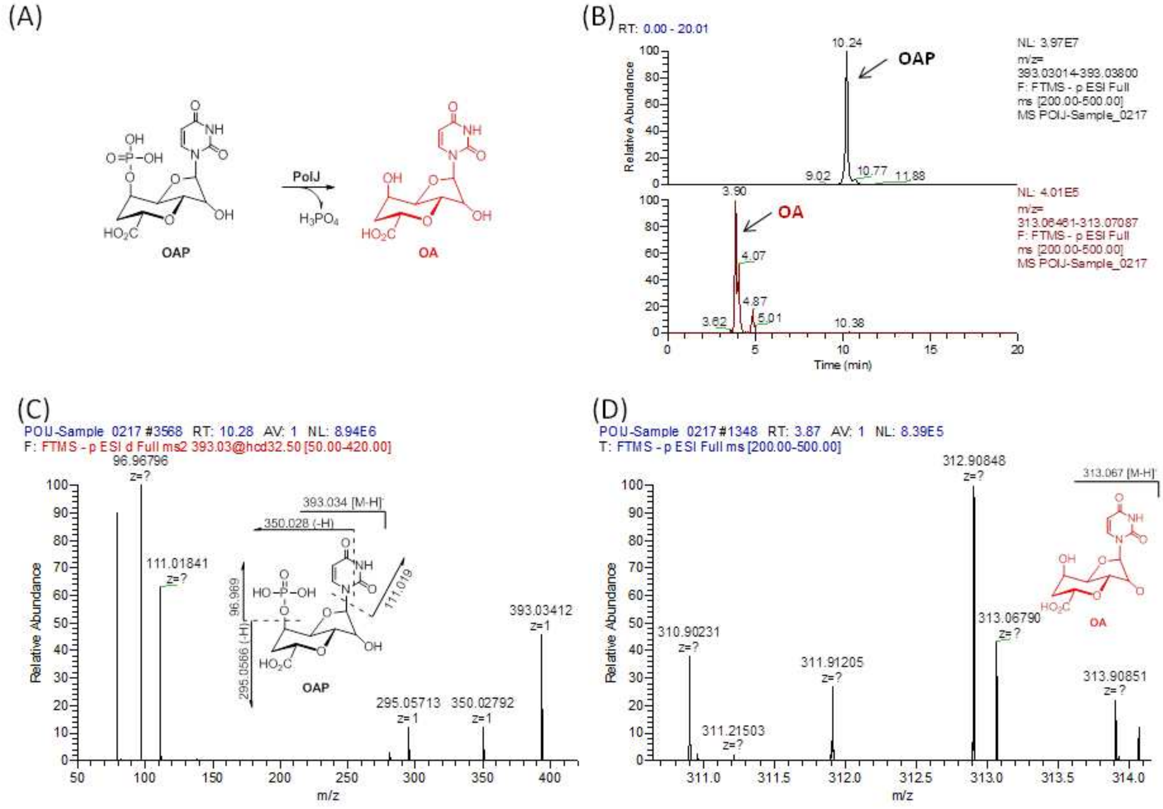

(A) PolJ reaction using OAP as substrate. The purified N-His$_6$-PoHJ was incubated with OAP (0.2 mM), $MgCl_2$ (1.0 mM) in 50 mM Tris-HCl buffer (pH 8.0) at 25°C for 8 hr.

(B) LC-MS/MS analysis of reaction mixture.

(C) Comparative tandem MS analysis of the substrate OAP (from left to right, 96.967, 111.018, 295.057, 350.027, 393.034)**.**

(D) Comparative tandem MS analysis of the product OA (from left to right, 310.902, 311.912, 312.908, 313.067, 313.908)**.**

Figure S25: The time course of PolH activity assay with the stoichiometric production of OAP and 5'-dA.

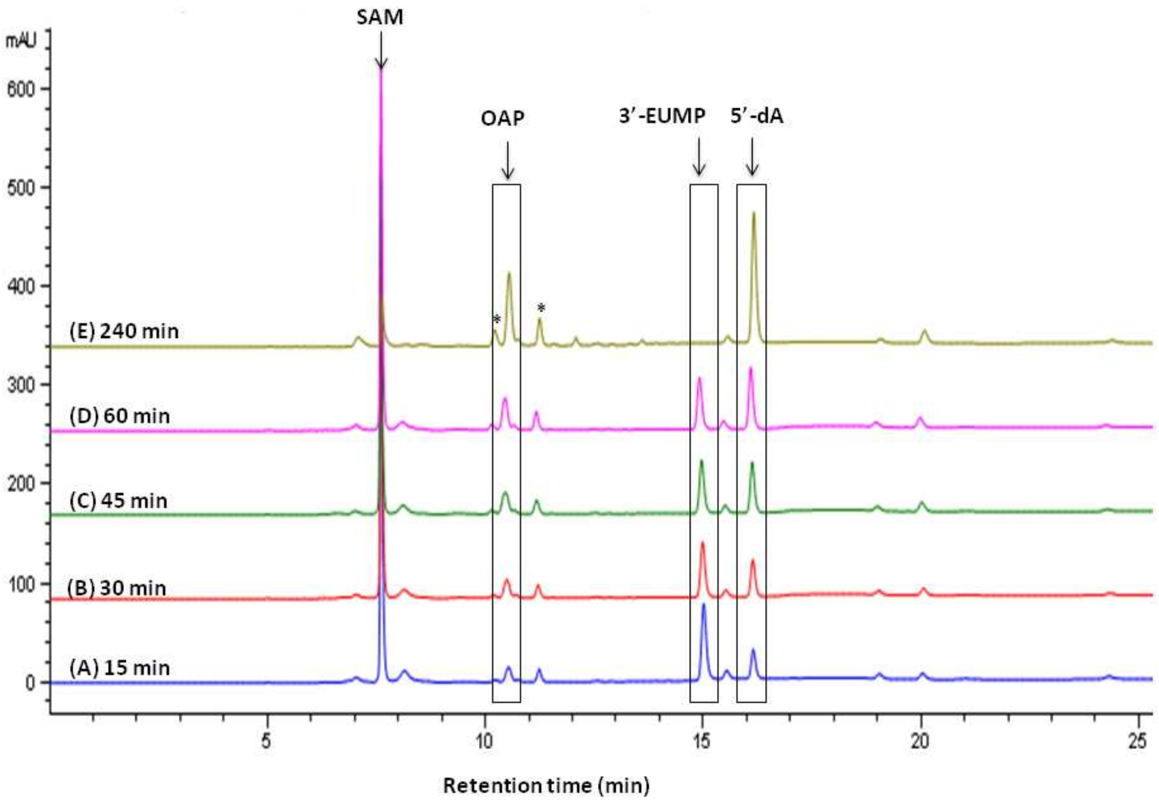

HPLC analysis of the PolH reaction by time courses with 3'-EUMP (0.5 mM), SAM (1.0 mM), sodium dithionite (2.0 mM) at rt anaerobically. (A) 15 min, (B) 30 min, (C) 45 min, (D) 60 min, (E) 240 min. The same HLPC condition as above PolH activity assay.

Figure S26: The PolH reaction in the 60% D_2O buffer solution.

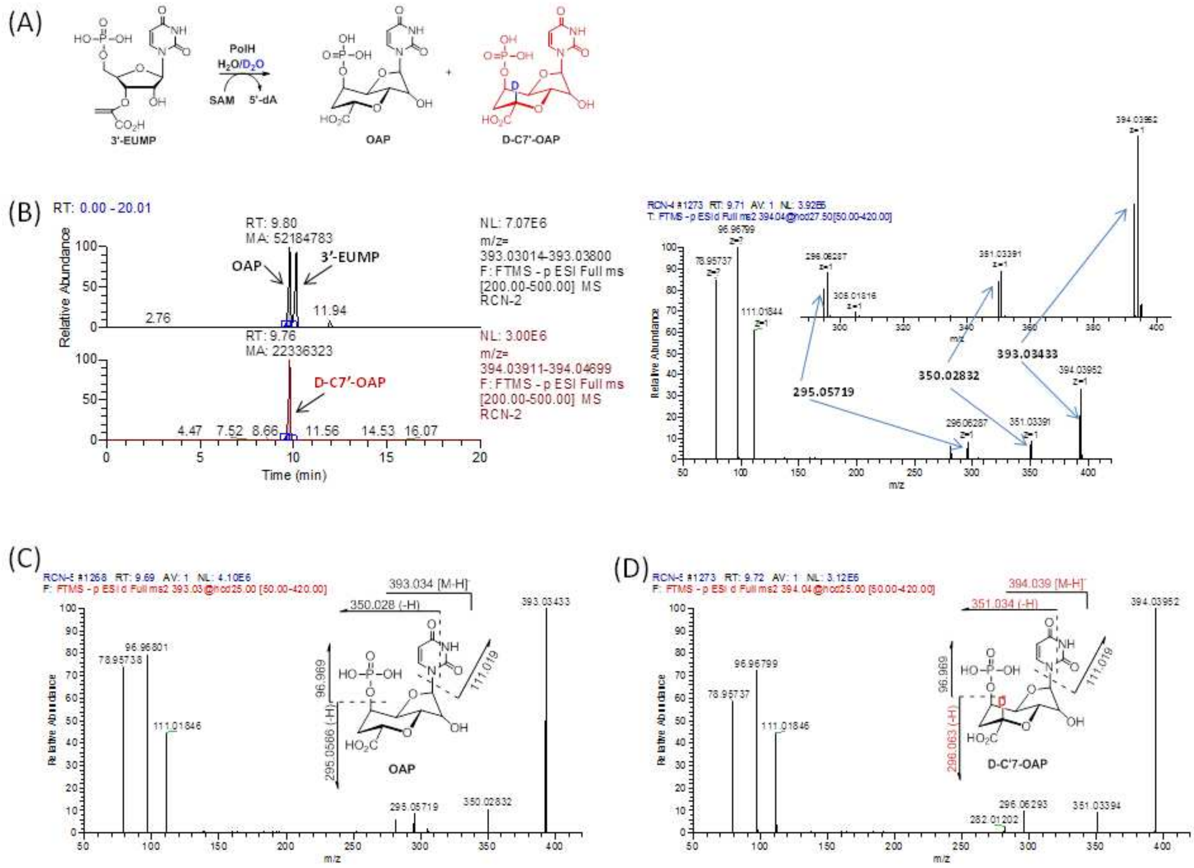

(A) The scheme of PolH reaction using 3'-EUMP as substrate in 60% D_2O buffer solution. Reconstituted PolH (20 μM) was incubated with 3'-EUMP (0.5 mM), SAM (1.0 mM), and dithionite (2.0 mM) at 25 ^{o}C in 50 mM Tris-HCl buffer (pH 8.0) containing 60% D_2O anaerobicaly.

(B) LC-MS/MS analysis of reaction mixture (B-2 (from left to right): 78.957, 96.967, 111.018, 295.057, 296.062, 350.028, 351.033, 393.034, 394.039).

(C) Comparative tandem MS analysis of the product OA (from left to right, 78.957, 96.967, 111.018, 296.062, 351.033, 394.039)**.**

(D) Comparative tandem MS analysis of the deuterium labeled product D-C7'-OA (from left to right, 78.957, 96.968, 111.018, 295.057, 350.028, 393.034)**.**

Scheme S1. PolJ and PolH reaction with 3'-EUMP as substrate.

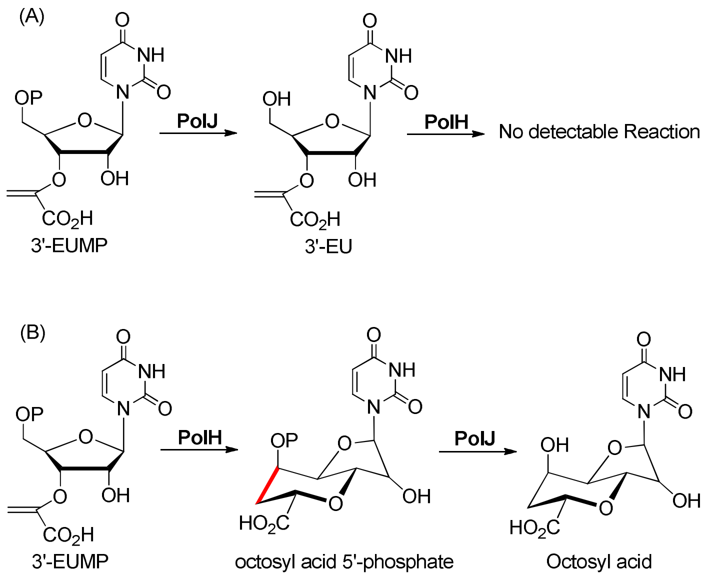

Table S1. Strains, plasmids, and cosmids used in this study.

Strain, plasmid, or cosmid	Relevant genotype or description	Source/Reference
S. aureochromoges		
YB172	Industrial polyoxin producer	Lab collection
CXR14	YB172 derivative with the entire polyoxin gene cluster deleted	This study
CXR14::4774	CXR14 derivative containing pJTU4774	This study
CXR14::4774/*ΔpolH*	CXR14 derivative containing pJTU4774/*ΔpolH*	This study
CXR14::4774/*ΔpolJ*	CXR14 derivative containing pJTU4774/*ΔpolJ*	This study
CXR14::2463	CXR14 derivative containing pJTU2463	This study
E. coli		
DH10B	F^- *mcr*A Δ(*mrr-hsd*RMS-*mcr*BC) ϕ80d *lacZ*Δ M15 Δ*lac*X74 *deo*R *rec*A1 *end*A1 *ara* Δ139 Δ(*ara leu*)1697 *gal*U *gal*Kλ *rps*L *nup*G	GIBCO BRL
BW25113	Δ*araBAD* Δ*rhaBAD*	[1]
ET12567	*rec*F *dam dcm hsd*S (Cml^R Km^R)	[2]
BL21(DE3)pLysE	F^- ,*ompT, hsdS$_B$ (r_B^- m_B^-) ,gal ,dcm* (DE3), pLysE (Cm^R)	Stratagene
**Fungi**		
Trichosporon cutaneum 3071	Indicator fungi for the bioassay of polyoxins as well as an opportunistic human fungal pathogen[3]	CGMCC[1]
**Plasmids/cosmids**		
pIJ790	λ-RED (*gam bet exo*) Cml^R *araC rep101*^{ts}	[4]
pOJ446	*aac (3)IV*, SCP2, *rep* MB1* *att*PϕC31 *ori*T	[5]
pBlueScriptII SK(+)	*bla lacZα ori*f1	Stratagene
pJTU2463	9 kb cosmid vector containing 3 λ *cos* sites; pOJ446 derivative, SCP2 replicon (3.5 kb EcoRI-XbaI) replaced by pSET152 *int att*PϕC31	(Yao et al. unpublished)
pJTU2178	*nikO* structural gene was cloned into NdeI-EcoRI sites of pET28a	
pJTU2876	pBlueScriptII SK(+) derivative with EcoRI-engineered *polH* structural gene amplified from 5A7 cloned into its SmaI-EcoRI sites	This study
pJTU2877	pET28a derivative with NdeI-EcoRI engineered fragment containing *polH* structural gene from pJTU2876cloned into counterpart sites of the vector	This study
pJTU2863	pBlueScriptII SK(+) derivative with EcoRI-engineered *polJ* structural gene amplified from pJTU4774 into its SmaI-EcoRI sites	This study
pJTU2864	pET28a derivative with NdeI-EcoRI engineered fragment containing *polJ* structural gene from pJTU2864 cloned into its counterpart sites	This study
pJTU4774	The cosmid with XbaI and SpeI sites infilled containing the entire polyoxin gene cluster	[6]
pJTU4774/*ΔpolH*	pJTU4774 derivative with *polH* was in frame deleted *via* PCR-targeting technology	This study
pJTU4774/*ΔpolJ*	pJTU4774 derivative with *polJ* was in frame deleted *via* PCR-targeting technology	This study
pJTU5701	22F7 with XbaI and NheI sites infilled and religated separately; no *nik* genes deleted	This study

*ori*T, origin of transfer of plasmid RK2; *tsr*, thiostrepton resistance gene; *aac(3) IV*, apramycin resistance gene; Cml^{R}, chloramphenicol resistance gene; Km^{R}, kanamycin resistance gene; [1]CGMCC, China General Microbiological Culture Collection Center.; rep^{pMB1*}, mutated rep^{pMB1}.

Table S2. PCR primers used in this study.

Primers	5' Sequence 3'
polHexF	ccatATGTCTGAGCACGTCACGC
polHexR	ggaattcTCACGATGCCCCCAGCAGCA
polJexF	ccataTGACCACCGGAGCCCGCC
polJexR	ggaattcTCAATCAGCGTCATGTCGTT
polHtgtF	GCCCTCTACCAGGTGGCCGGTCACCTGCGCACCGCGCTG TCTAGAGCTATTCCAGAAGT
polHtgtR	GACGGTCGCGATGGTGTGCCGCACCCCCTCCACGCTGGT ACTAGTCTGGATGCCGACG
polJtgtF	AACGTCCGCGACAGCGCCGGGCTGCCCGGCCCGGACGGC TCTAGAGCTATTCCAGAAGT
polJtgtR	GACGCTCTCGTGTTCGTCCTCGATGCCGGTGATCAGGCT ACTAGTCTGGATGCCGACG

Table S3. Comparative analysis of the target *pol* genes and their homogs involved in potential AHA biosynthetic pathway

**Protein (aa)**	**Proposed Function**	***S. tendae* Tü 901,** Homolog (aa), Identity, Similarity, (%)	***S. chromofuscus* ATCC49982** Homolog (aa) , Identity, Similarity, (%)	***S. aurantiacus* JA4570** Homolog (aa) , Identity, Similarity, (%)	***Xenorhabdus szentirmaii* DSM16338**, Homolog (aa), Identity, Similarity, (%)
PolA (445)	UMP enolpyruvyl transferase	NikO (473) (63 ,73)	sccontig008-75 (495) (40,53)	STRAU_0242 (423) (33,47)	XSR1_370018 (436) (35,57)
PolD (215)	Hydroxylase	NikI (218) (50, 66)	sccontig008-76 (201) (28,46)	STRAU_0246 (216) (53,67)	XSR1_370026 (221) (31,48)
PolH, (469)	Radical SAM protein	NikJ (454) (70, 81)	sccontig008-80 (465) (52,63)	STRAU_0239 (436) (51,63)	XSR1_370015 (447) (43,60)
PolI (380)	Aminotransferase	NikK (368) (62, 70)	sccontig008-81 (392) (35,47)	STRAU_0238 (374) (32,44)	XSR1_370019 (371) (27,47)
PolJ (273)	Phosphatase	NikL(240) (61, 69)	sccontig008-78 (242) (31,48)	STRAU_0240 (223) (35,49)	XSR1_370020 (231) (30,46)
PolK (213)	Hydroxylase	NikM (213) (58, 70)	sccontig008-74 (240) (47,59)	STRAU_0236 (207) 36,53	XSR1_370017 (206) 35,48

4. Methods and Experimental section

Method S1. Materials, instruments and general methods.

All chemicals and reagents were purchased from Sigma-Aldrich (USA) or Aladdin (China) unless otherwise stated. Strains, plasmids and primers used in this study were independently listed in Table S1and Table S2. General methods were according to the standard methods of Sambrook et al.[7] or Kieser et al.[8] The final antibiotic concentrations used in this study were as follows: 100 μg/ml ampicillin, 30 μg/ml apramycin, 50 μg/ml kanamycin, 25 μg/ml chloramphenicol and 12.5 μg/ml thiostrepton. NMR spectra were recorded on a 500 MHz instrument (Bruker). The tandem MS of activity assay and digested protein was detected on the Q Exactive Orbitrap Mass Spectrometry (Thermo) in the core facility center of SIPPE. The LC condition of LC-MS/MS was conducted using an analytical Phenomenex Luna NH_2 column (3 μM, 4 × 100 mm). The sample was eluted with a gradient of solvent A (50 mM NH_4OAC, pH 9.5) and solvent B (acetonitrile). The gradient was run from 30% A over 1 min, 30 to 95% A over 8 min, 95% A over 3 min, then 0.1 min at 95−30% A, and then re-equilibrated at 30% A for 8 min. The flow rate was 0.3 mL/min at 15 ^{o}C. The peaks corresponding to the enzymatic reaction products were subjected to ESI-MS or MS/MS analyses. EPR spectra were recorded using a Bruker EMX Plus 10/12 spectrometer (Bruker Co., Ltd., Germany) equipped with an Oxford ESR910 liquid helium continuous flow cryostat (Oxford Instrument Co., Ltd., UK) at the National High Magnetic Field Laboratory of the Chinese Academy of Sciences, Hefei, China. Acquisition conditions for the Fe-S cluster analysis are: microwave frequency, 9.39 GHz; microwave power, 1 mW; field modulationamplitude, 5 Gauss; modulation frequency, 100 kHz; temperature, 30 K.

Method S2. In frame deletion of *polH* or *polJ* in cosmid pJTU4774 by PCR-targeting technology.

For the targeted inactivation of *polH,* a kanamycin resistance cassette (*neo*) from SuperCos1 amplified using the primers polHtgtF and polHtgtR (Table S2), was recombined into pJTU5701 by λ-Red-mediated recombination[4] to give pJTU4774/*polH-neo*. The *neo* cassette was then deleted *in vitro* by XbaI and SpeI digestion (unique sites) and religated to produce pJTU4774/*ΔpolH*. The unmarked deletion was confirmed by PCR using primers polHexpF/R. Likewise, *polJ* in pJTU4774 was inactivated using the primers polJtgtF/R for the the targeted gene replacement, and polJexpF/R to confirm the unmarked deletion in pJTU4774/*ΔpolJ*.

Method S3. Cultivation and metabolites analysis of the *S. aureochromogenes* and its derivatives .

S. aureochromogenes and its derivatives were cultivated in liquid fermentation medium.[9] After the fermentation, the broth was adjusted to pH 3.0 with oxalic acid and filtered for LC-MS analysis and targeted metabolites preparation. Bioassay of the metabolites was based on the procedures by Zhai et al.[6]

For the detection of the target metabolites accumulated by related *S. aureochromogenes* and its derivatives, the HPLC (Shimadzu LC-20A) analysis were performed on a ZORBAX SB-C18 column (Agilent, 3.5 mm, 4.6×250 mm) with an elution gradient of 5%-55% methanol: 0.15% aqueous TFA (HPLC grade) over 30 min at a flow rate of 0.5 mL/min, and then keep the ratio of 55% methanol : 0.15% aqueous TFA for another 30 min at 0.5 mL/min. The elution was monitored at UV 263 nm with DAD detector.

For the purification of the target metabolite (**Compound X**), the conditions are the identical to the above except the preparation was performed on a ZORBA SB-C18 Column (Agilent, 5 μm, 9.4×250 mm) at flow rate of 3 mL/min. The purified metabolite was subsequently concentrated by vaccum, after that, the identity of the metabolite was preliminarily confirmed by LC-MS analysis (Thermo LTQ-Orbitrap) with positive ion mode, and the detailed parameters are as follows: drying gas temperature: 275 °C, drying gas flow: 10 liters/mL, and nebulizer pressure: 30 p.s.i.

Method S4. Preparation of the NikO-catalyzed product, 3'-EUMP.

NikO was overexpressed and purified based on the procedures described previously.[9] After the reactions, the NikO protein was inactivated by adding equal volume of methanol, and removed by centrifuging at 12,000 r/m for 10 min. Subsequently, the 100 mL supernant of the reactions was concentrated by vaccum to volum of 5 ml, then 3'-EUMP was prepared and its identity was confirmed by LC-MS (in negative ion mode) and NMR analysis. The HPLC preparation was conducted on a ZORBA SB-C18 Column (Agilent, 5 μm, 9.4×250 mm) with an elution gradient of 5%-30% methanol: 0.3% aqueous TFA (HPLC grade) over 20 min at a flow rate of 3.0 mL/min, and then keep the ratio 30% methanol: 0.3% aqueous TFA for another 20 min at 3.0 mL/min. The elution was monitored at UV 263 nm with a DAD detector.

Method S5. Cloning of PolH and PolJ in *E. coli.*

For the construction of the *polH* and *polJ* expression vector, the structural gene of them were amplified with individual pairs of the primers polHexF/polHexR and polJexF/polJexR. The EcoRI-engineered PCR products were then cloned into SmaI-EcoRI sites of pBlueScriptII SK(+) to generate pJTU2876/pJTU2863. After confirmation by DNA sequencing, the NdeI-EcoRI engineered fragments containing both structural genes from the plasmids were cloned into the counterpart sites of pET28a to give pJTU2877 and pJTU2864, respectively.

Method S6. Over-expression and purification of PolJ.

A culture of *E. coli* BL21(DE3) cells harboring the PolJ/pET28a construct were grown in the LB medium containing 50 μg/mL of kanamycin at 37 °C with shaking (200 rpm) until the OD_{600} reached ~0.5. Protein expression was then induced by addition of isopropyl β-D-1-thiogalactopyranoside (IPTG) to a final concentration of 0.5 mM, and the cells were allowed to grow at 18 °C and 200 rpm for an additional 18 h. The cells were harvested by centrifugation at 4500 × g for 15 min. All purification steps were carried out

at 4 ^{o}C using Ni-NTA resin and the PolJ protein were washed and eluted by 20-250 mM imidazole in 25 mM Tris-HCl, 150 mM NaCl buffer (pH 8.0). The collected protein solution was dialyzed against 25 mM Tris-HCl, 150 mM NaCl buffer (pH 8.0) for three times. The protein solution was then flash-frozen in liquid nitrogen and stored at -80 ^{o}C until use. Protein concentration was determined by the Bradford assay using bovine serum albumin as the standard.

Method S7. PolJ activity assays.

The PolJ activity assays were performed with the purified N-His$_6$-PoHJ incubated with OAP (0.2 mM) or 3'-EUMP (1.0 mM), $MgCl_2$ (1.0 mM) in 50 mM Tris-HCl buffer (pH 8.0) at 25°C for 8 hr. The reaction mixture was quenched with equal volume amount of MeOH and centrifuged to remove protein precipitation then filtrated with 0.22 µM of filter before injecting to HPLC. The assay was monitored by LC-MS/MS with the general method by the Q Exactive Orbitrap Mass Spectrometry.

Method S8. Purification and reconstitution of PolH protein.

Following the similar procedure as the over-expression and purification of PolJ, PolH was purified anaerobically in the Coy-Chamber glove box from the *E. coli* BL21(DE3)/plysE cells harboring the PolH/pET28a construct in 4 ^{o}C.

The reconstitution was performed by incubation of as-isolated PolH with 6 equal excess mole amount of Na_2S, $Fe(NH_4)_2(SO_4)_2$ in presence of 5.0 mM DTT for 3 hr at 4 ^{o}C anaerobically. Then the reconstituted solution was desalted by G-10 column then washed with the buffer (25 mM Tris-HCl, 150 mM NaCl, 5% glycerol buffer, pH 8.0). The fractions contained PolH were collected and concentrated by ultrafiltration.

Method S9. PolH activity assays

The PolH activity assays were performed at 37°C or room temperature under anaerobic conditions for a proper time in a Coy-chamber glove box. The reaction mixture contained the following: reconstituted PolH (20 µM), 3'-EUMP (0.5 mM), SAM (1.0 mM) and sodium dithionite (2.0 mM) in 50 mM Tris-HCl (pH 8.0) anaerobically. The reaction mixture was quenched with equal volume amount of MeOH and centrifuged to remove protein precipitation then filtrated with 0.22 µM of filter before injecting to HPLC.

The reaction conversion was monitored in the Agilent 1260 HPLC equipped the phenomenex luna C18 column (5.0 µM, 250 mm x 4.6 mm) with a gradient condition of solvent A (MeOH with 0.15% TFA) and solvent B (0.15% TFA in H_2O) below. The gradient was run from 5% to 30% A over 10 min, 30% to 50% A over 15 min, 30% to 50% A over 1 min , then 4.0 min at 50−5% A, and then re-equilibrated at 5% A for 5 min. The flow rate was 0.5 mL/min at 35 ^{o}C.

The product was purified with the HPLC condition above. The LC-MS/MS was detected with the general method by the Q Exactive Orbitrap Mass Spectrometry.

5. Supplementary References.

Reference S1. Supplementary References.

1. Datsenko, K. A.; Wanner, B. L., *Proc. Natl. Acad. Sci. USA* **2000,** *97*, 6640.

2. Paget, M. S.; Chamberlin, L.; Atrih, A.; Foster, S. J.; Buttner, M. J. *J. Bacteriol.* **1999**, *181*, 204.

3. Lowenthal, R. M.; Atkinson, K.; Challis, D. R.; Tucker, R. G.; Biggs, J. C., *Bone Marrow Transplant* **1987,** *2*, 321

4. Gust, B.; Challis, G. L.; Fowler, K.; Kieser, T.; Chater, K. F., *Proc. Natl. Acad. Sci. USA* **2003,** *100*, 1541

5. Bierman, M.; Logan, R.; O'Brien, K.; Seno, E. T.; Rao, R. N.; Schoner, B. E., *Gene* **1992,** *116* (1), 43-9.

6. Zhai, L.; Lin, S.; Qu, D.; Hong, X.; Bai, L.; Chen, W.; Deng, Z., *Metab. Eng.* **2012,** *14*, 388.

7. Sambrook, J.; Fritsch, E. F.; Maniatis, T., *Molecular Cloning: a Laboratory Manual* **1989**, 2nd ed., Cold Spring Harbor Laboratory Press, NY.

8. Kieser, T.; Bibb, M. J.; Chater, K. F.; Butter, M. J.; Hopwood., D. A., *Practical Streptomyces Genetics* **2000**, 2nd ed., John Innes Foundation, Norwich, United Kingdom.

9. Chen, W.; Huang, T.; He, X.; Meng, Q.; You, D.; Bai, L.; Li, J.; Wu, M.; Li, R.; Xie, Z.; Zhou, H.; Zhou, X.; Tan, H.; Deng, Z., *J. Biol. Chem.* **2009,** *284*, 10627.
